# Targeting tRNA‐Derived Non‐Coding RNA Alleviates Diabetes‐Induced Visual Impairment through Protecting Retinal Neurovascular Unit

**DOI:** 10.1002/advs.202411042

**Published:** 2024-11-08

**Authors:** Jin Yao, Wen Yao, Jun‐Ya Zhu, Yan Liu, Jin‐Hong Liu, Yu‐Ke Ji, Xi‐Shen Ni, Wan Mu, Biao Yan

**Affiliations:** ^1^ The Affiliated Eye Hospital Nanjing Medical University Nanjing 210000 China; ^2^ School of Medicine Southeast University Nanjing 210009 China; ^3^ Department of Ophthalmology Shanghai General Hospital Shanghai Jiao Tong University School of Medicine Shanghai 200080 China; ^4^ Eye Institute and Department of Ophthalmology Eye and ENT Hospital Fudan University Shanghai 200031 China

**Keywords:** diabetic retinopathy, neuropathy, neurovascular dysfunction, tRNA‐derived stress‐induced RNAs, vasculopathy

## Abstract

Diabetes is a major risk factor for compromised visual health, leading to retinal vasculopathy and neuropathy, both of which are hallmarks of neurovascular unit dysfunction. Despite the critical impact of diabetic retinopathy, the precise mechanism underlying neurovascular coupling and effective strategies to suppress neurovascular dysfunction remain unclear. In this study, the up‐regulation of a tRNA‐derived stress‐induced RNA, 5′tiRNA‐His‐GTG, in response to diabetic stress is revealed. 5′tiRNA‐His‐GTG directly regulates Müller glia action and indirectly alters endothelial angiogenic effects and retinal ganglion cell (RGC) survival in vitro. Downregulation of 5′tiRNA‐His‐GTG alleviates diabetes‐induced retinal neurovascular dysfunction, characterized by reduced retinal vascular dysfunction, decreased retinal neurodegeneration, and improved visually‐guided behaviors in vivo. Mechanistically, 5′tiRNA‐His‐GTG acts as a key regulator of retinal neurovascular dysfunction, primarily by modulating arachidonic acid (AA) metabolism via the CYPs pathway. The 5′tiRNA‐His‐GTG‐CYP2E1‐19(S)‐HETE signaling axis is identified as a key driver of retinal neurovascular dysfunction. Thus, targeting 5′tiRNA‐His‐GTG presents a promising therapeutic strategy for treating vasculopathy and neuropathy associated with diabetes mellitus. Modulating this novel signaling pathway can open up new avenues for intervention in diabetic retinopathy and its related complications.

## Graphical abstract

This study reveals that 5'tiRNA‐His‐GTG, a stress‐induced non‐coding RNA, worsens diabetic retinal neurovascular impairment by modulating arachidonic acid metabolism via CYP2E1‐19(S)‐HETE pathway. Targeting 5'tiRNA‐His‐GTG reduces retinal vascular and neuronal damage, promoting visual function recovery. This study identifies a novel 5'tiRNA‐His‐GTG‐mediated signaling axis as a promising therapeutic target for diabetic retinopathy.

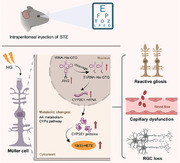

## 1 Introduction

Diabetes mellitus (DM) is a hyperglycemic metabolic disease arising from deficiencies in insulin secretion or resistance.^[^ ^1^ ^]^ Its global prevalence is notably high and anticipated to rise to 783 million by 2045.^[^ ^2^ ^]^ Long‐term hyperglycemia induces damage and dysfunction in various organs, especially the heart, blood vessels, nerves, eyes, and kidneys.^[^ ^3^, ^4^ ^]^ Severe vascular complications, including cardiovascular disease, nephropathy, and retinopathy, are major contributors to blindness, kidney failure, and elevated mortality in diabetic patients.^[^ ^5^ ^]^ Additionally, nearly half of DM patients experience diabetic neuropathy involving peripheral, autonomic, and cranial nerves, with symptoms such as pain and decreased motility.^[^ ^6^, ^7^ ^]^ DM adversely affects the normal functioning of both nervous and vascular systems. Currently, therapeutic strategies primarily concentrate on glycemic control, alongside adopting a healthy lifestyle and managing related complications with additional medications.^[^ ^8^, ^9^ ^]^ Nevertheless, these interventions cannot completely prevent the neurological and vascular pathological changes stemming from prolonged glucose metabolic disorders. Consequently, there is an urgent need for therapeutic methods designed to prevent and treat vasculopathy and neuropathy associated DM.

Epigenetics refers to a heritable trait that affects gene activity without any changes in DNA sequence.^[^ ^10^ ^]^ Upon diabetic stress, shifts in environmental factors, such as nutrients, inflammatory mediators, and hormones, induce major changes in the epigenetic mechanism of various cells.^[^ ^11^ ^]^ Small non‐coding RNAs are present in most organisms and serve various functions, encompassing microRNAs (miRNAs), piwi‐interacting RNAs (piRNAs), small nucleolar RNAs (snoRNAs), and a new class of tRNA‐derived small RNAs (tsRNAs).^[^ ^12^, ^13^ ^]^ Within the category of tsRNAs, tRNA‐derived stress‐induced RNAs (tiRNAs) are 30–45 nt products, resulting from the cleavage of angiogenin (ANG) in the anticodon loops of tRNAs under diseased conditions, such as starvation, oxidative stress, or hypoxia.^[^ ^14^ ^]^ tiRNAs display a range of biological functions, including influencing protein synthesis, regulating gene expression, stabilizing mRNA, and participating in stress response.^[^ ^15^, ^16^ ^]^ tiRNAs are involved in the pathogenesis of several human diseases, including metabolic disorders, inflammation, tumors, and neurological diseases.^[^ ^17^, ^18^ ^]^ Moreover, tiRNAs have emerged as potential biomarkers and modulators for disease diagnosis and prognosis.^[^ ^19^, ^20^ ^]^ However, the role of tiRNAs in DM remains at an early stage of research.

Diabetic retinopathy (DR), one of the most prevalent diabetic complications, stands as a major contributor to vision loss and blindness among working‐age adults.^[^ ^21^ ^]^ DR is often classified as a microvascular disorder, which is characterized by damage to tight junctions, pericyte loss, and thickening of endothelial basal membrane.^[^ ^22^ ^]^ Notably, the retina, an integral part of the central nervous system (CNS), is a highly specialized tissue.^[^ ^23^ ^]^ Emerging evidence suggests that neurodegeneration occurs in the pathogenesis of DR, involving alterations in both neurons and glial cells.^[^ ^24^ ^]^ Thus, DR is increasingly recognized as a neurovascular complication caused by the disruptions of retinal neurovascular unit (NVU), extending beyond a mere microvascular disease.^[^ ^25^ ^]^ Comprising neurons, glial cells, and blood cells, retinal NVU plays a crucial role in regulating blood‐retinal barrier and synaptic connections among retinal neurons.^[^ ^26^ ^]^ s NVU dysfunction contributes to the pathogenesis of DR, featuring prominent pathological features, such as retinal neurodegeneration, inflammation, and microangiopathy.^[^ ^27^ ^]^ However, the precise mechanisms through which NVU influences the progression of DR remain to be fully elucidated.

In this study, we have identified that 5′tiRNA‐His‐GTG plays a pivotal role in the development of retinal neurovascular impairment induced by diabetes. The expression level of 5′tiRNA‐His‐GTG was significantly up‐regulated upon diabetes‐related stresses. Notably, the attenuation of 5′tiRNA‐His‐GTG expression could ameliorate DR‐induced retinal dysfunction, characterized by the mitigation of retinal vascular dysfunction and the suppression of retinal neurodegeneration. Mechanistically, 5′tiRNA‐His‐GTG regulated retinal neurovascular dysfunction through the modulation of AA metabolism via the CYPs pathway. The signaling axis involving 5′tiRNA‐His‐GTG, CYP2E1, and 19(S)‐HETE has been identified as a contributor to retinal neurovascular dysfunction. Overall, targeting 5′tiRNA‐His‐GTG offers promising potential as a therapeutic strategy for addressing vasculopathy and neuropathy associated with DM.

## 2 Results

### 2.1 Expression Pattern of 5′tiRNA‐His‐GTG is Up‐Regulated under Diabetic Condition

**Figure 1:**
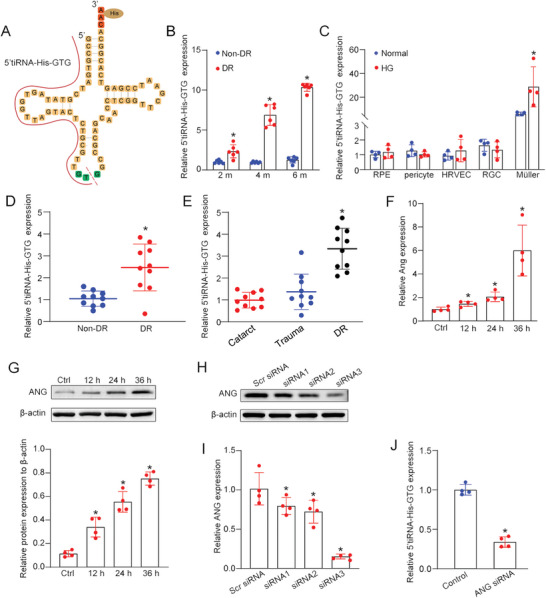
Identification of 5′tiRNA‐His‐GTG as a high glucose‐regulated circRNA in Müller cells. A) Structure of 5′tiRNA‐His‐GTG and mature tRNA‐His‐GTG. B) qRT‐PCR assays were performed to detect the expression of 5′tiRNA‐His‐GTG in non‐diabetic retinas and diabetic retinas following 2‐, 4‐, or 6‐month of STZ injection (n = 6, *p < 0.05, Student's *t‐*test). C) 5′tiRNA‐His‐GTG expression was examined by qRT‐PCRs in retinal pigment epithelium(RPE), pericytes, human retinal vascular endothelial cells (HRVECs), primary retinal ganglion cells (RGCs), and primary Müller cells were exposed to high glucose (HG, 30 mM), or left untreated (Ctrl) for 48 h (n = 4, *p < 0.05, Student's *t‐*test). D) The expression of 5′tiRNA‐His‐GTG in fibrovascular membranes from diabetic patients and idiopathic epiretinal membranes from non‐diabetic patients were determined using qRT‐PCR assays (n = 10, *p < 0.05, Student's *t‐*test). E) The expression of 5′tiRNA‐His‐GTG in aqueous humor (AH) from patients with trauma, cataract, and DR were determined by qRT‐PCR assays (n = 10, *p < 0.05, one‐way ANOVA followed by post hoc Bonferroni test). F) qRT‐PCRs were performed to detect ANG expression in Müller cells following high glucose treatment (HG, 30 mM) for 12 h, 24 h, and 36 h (n = 4, *p < 0.05, one‐way ANOVA followed by post hoc Bonferroni test). G) Western blots and quantitative analysis were conducted to detect the protein levels of ANG in Müller cells following 12 h, 24 h, and 36 h of high glucose treatment (n = 4, *p < 0.05, one‐way ANOVA followed by post hoc Bonferroni test). H,I) Müller cells were transfected with ANG siRNA‐1, 2, 3, or negative control (NC) siRNA for 48 h. ANG protein level was detected by western blots (H, n = 4). ANG mRNA level was detected by qRT‐PCR assays (I, n = 4, *p < 0.05, one‐way ANOVA followed by post hoc Bonferroni test). J) Müller cells were transfected with control (negative control, NC) siRNA or ANG siRNA for 6 h. Total RNAs were isolated and 5′tiRNA‐His‐GTG level was detected by qRT‐PCR assays (n = 4, *p < 0.05, one‐way ANOVA followed by post hoc Bonferroni test).

5′tiRNA‐His‐GTG is a transcript derived from 5′ half of mature tRNA‐His‐GTG (**Figure** **1**A). To explore the expression pattern of 5′tiRNA‐His‐GTG under diabetic condition in vivo and in vitro. Eight‐week‐old C57BL/6J mice were injected with streptozotocin (STZ) for 5 consecutive days to establish a diabetic model. qRT‐PCR assays revealed that retinal 5′tiRNA‐His‐GTG expression was higher in diabetic mice compared to non‐diabetic controls, increasing with diabetes progression over time (Figure 1B). To address the role of 5′tiRNA‐His‐GTG in various retinal cells, primary Müller cells, human retinal microvascular endothelial cells (HRMECs), retinal ganglion cells (RGCs), pericytes, and retinal pigment epithelium cells (RPEs) were exposed to high glucose to mimic diabetic stress in vitro. qRT‐PCR assays showed that 5′tiRNA‐His‐GTG expression was significantly up‐regulated in Müller cells following diabetic stress in vitro (Figure 1C). To reveal the expression pattern of 5′tiRNA‐His‐GTG in clinical samples, we collected fibrovascular membranes from DR patients and idiopathic epiretinal membranes from non‐diabetic patients. Compared with non‐diabetic control patients, the level of 5′tiRNA‐His‐GTG expression was up‐regulated in fibrovascular membranes of DR patients (Figure 1D). Additionally, elevated levels of 5′tiRNA‐His‐GTG were observed in aqueous humor (AH) of DR patients compared to those with cataracts or trauma (Figure 1E). These findings collectively suggest that 5′tiRNA‐His‐GTG may play a significant role in the progression of DR.

tiRNA is known to be generated by the cleavage of ribonuclease ANG in the anticodon loops of mature tRNA.^[^ ^28^ ^]^ To validate the origin of 5′tiRNA‐His‐GTG, we conducted qRT‐PCR assays and western blots, which revealed a significant up‐regulation of both ANG mRNA and protein levels in Müller cells subjected to high‐glucose stress (Figure 1F,G). Next, Müller cells were transfected with three different small interfering RNAs (siRNAs) for 48 h to reduce ANG expression. Western blots and qRT‐PCR assays revealed that siRNA3 exhibited the highest silencing efficiency (Figure 1H,I). To further assess whether the generation of 5′tiRNA‐His‐GTG is mediated by ANG, qRT‐PCR assays were conducted, revealing a decrease in 5′tiRNA‐His‐GTG expression following ANG knockdown (Figure 1J). These findings suggest that 5′tiRNA‐His‐GTG is likely produced through ANG‐mediated cleavage.

### 2.2 5′tiRNA‐His‐GTG Regulates Retinal Vascular Function and Neuronal Function In Vitro

**Figure 2:**
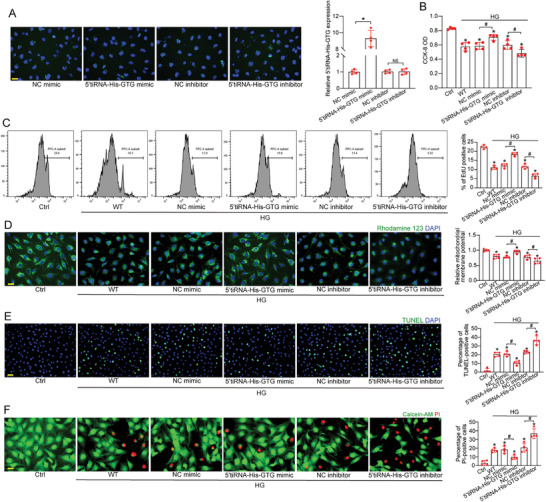
The effect of 5′tiRNA‐His‐GTG on Müller cell function under diabetic condition in vitro. A) Primary Müller cells were transfected with FAM‐labeled negative control (NC) mimic, 5′tiRNA‐His‐GTG mimic, NC inhibitor, or 5′tiRNA‐His‐GTG inhibitor (green dots) for 24 h. Fluorescent images of Müller cells were observed by a fluorescence microscopy. Meanwhile, the levels of 5′tiRNA‐His‐GTG were determined by qRT‐PCRs. Scale bar, 20 µm (n = 4, *p < 0.05, NS, no significant difference, one‐way ANOVA followed by Bonferroni test). B – F) Primary Müller cells were transfected with NC mimic, 5′tiRNA‐His‐GTG mimic, NC inhibitor, and 5′tiRNA‐His‐GTG inhibitor, or left untreated (WT) for 6 h, and then exposed to high glucose (HG, 30 mM) for 48 h. The group exposed to 5.5 mM glucose was taken as the control (Ctrl) group. CCK‐8 assays were performed to evaluate cell viability (B, n = 4). Quantification of proliferating cells was performed by FACS analysis (C, n = 4). Rhodamine 123 staining was performed to detect the change of mitochondrial membrane potential. Rhodamine 123, green; DAPI, blue. Scale bar, 20 µm (D, n = 4). TUNEL assays were performed to detect apoptotic Müller cells. TUNEL, green; DAPI, blue. Scale bar, 50 µm (E, n = 4). Calcein‐AM/PI staining was performed to detect the dead or dying cells. Calcein‐AM, green; PI, red. Scale bar, 20 µm (F, n = 4). *p < 0.05 versus Ctrl; ^#^ *p* < 0.05 between the marked groups. The significant differences were evaluated by one‐way ANOVA followed by post hoc Bonferroni test.

Given 5′tiRNA‐His‐GTG was the most markedly up‐regulated transcript in Müller cells under high glucose condition in vitro, we investigated the role of 5′tiRNA‐His‐GTG in Müller cells. Primary Müller cells were isolated, and immunostaining for GS and GFAP confirmed a purity of over 95% (Figure S1, Supporting Information). To explore the biological effects of 5′tiRNA‐His‐GTG, fluorescence‐labeled negative control (NC) mimics, NC inhibitors, 5′tiRNA‐His‐GTG mimics, or 5′tiRNA‐His‐GTG inhibitors were transfected into Müller cells. The efficiency of overexpression and knockdown is shown in **Figure** **2**A. CCK‐8 assays demonstrated that the viability of Müller cells was decreased upon high glucose stress. Up‐regulation of 5′tiRNA‐His‐GTG enhanced the viability of Müller cells, while down‐regulation of 5′tiRNA‐His‐GTG reduced cell viability (Figure 2B). To determine whether 5′tiRNA‐His‐GTG affects the proliferative capacity of Müller cells, we conducted flow cytometry using a commercial EdU kit. The results indicated that up‐regulation of 5′tiRNA‐His‐GTG promoted Müller cell proliferation under high glucose condition, whereas its knockdown inhibited proliferation (Figure 2C). Rhodamine 123 staining assays were conducted to detect the effects of 5′tiRNA‐His‐GTG expression changes on mitochondrial membrane potential in Müller cells. The results showed that mitochondrial membrane potential was higher in the 5′tiRNA‐His‐GTG mimic group compared to the NC mimic group (Figure 2D). Conversely, transfection with 5′tiRNA‐His‐GTG inhibitor resulted in a decreased mitochondrial membrane potential. TUNEL and PI/Calcein‐AM double staining indicated that 5′tiRNA‐His‐GTG up‐regulation mitigated high glucose‐induced Müller cell apoptosis, as shown by fewer TUNEL and PI‐positive cells, while the inhibitor displayed the opposite effect (Figure 2E,F).

As Müller cells spanning the entire retina and enveloping retinal blood vessels and neurons, they facilitate intimate neurovascular communication.^[^ ^29^, ^30^ ^]^ To study the interactions between endothelial cells (ECs)‐glia and neuron‐glia, Müller cells were co‐cultured with HRMECs or RGCs under high glucose condition in vitro. The results demonstrated that co‐culture with Müller cells contributed to pro‐angiogenic effects, enhancing HRMEC proliferation, migration, and tube formation. However, the down‐regulation of 5′tiRNA‐His‐GTG in Müller cells partially reversed these pro‐angiogenic effects (Figure S2, Supporting Information).

RGCs were then isolated from C57BL/6J mouse pups. TUJ1 immunostaining revealed RGC purity exceeded 85% (Figure S3A, Supporting Information). CCK‐8 and TUNEL assays demonstrated co‐culture with Müller cells reduced RGC viability and survival when exposed to high glucose for 24 h. Transfection of 5′tiRNA‐His‐GTG mimic into Müller cells exacerbated high glucose‐mediated RGC damage (Figure S3B,C, Supporting Information). Collectively, these results demonstrate that 5′tiRNA‐His‐GTG indirectly regulates vascular and neuronal function through direct regulation of Müller cell activity.

### 2.3 5′tiRNA‐His‐GTG Regulates Diabetes‐Induced Retinal Vascular Dysfunction In Vivo

**Figure 3:**
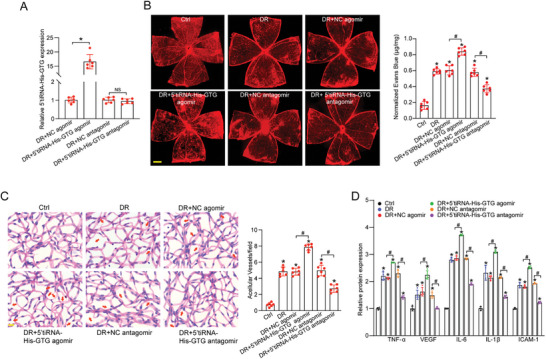
The effect of 5′tiRNA‐His‐GTG on diabetes‐induced retinal vascular dysfunction in vivo. A) Diabetic C57BL/6J mice received intravitreal injections of negative control (NC) agomir, 5′tiRNA‐His‐GTG agomir, NC antagomir, or 5′tiRNA‐His‐GTG antagomir twice a month. The levels of 5′tiRNA‐His‐GTG were determined by qRT‐PCRs (n = 6, *p < 0.05, NS, no significant difference, one‐way ANOVA followed by Bonferroni test). B – D) Diabetic C57BL/6J mice received intravitreal injections of NC agomir, 5′tiRNA‐His‐GTG agomir, NC antagomir, or 5′tiRNA‐His‐GTG antagomir or left untreated (DR) twice a month. The non‐diabetic C57BL/6J mice were taken as the control (Ctrl) group. 4 × objective lens fluorescent microscope images of Evans Blue Injected flat‐mounted retinas. Evans blue leakage was quantified for blood vessel leakage following 4‐month diabetes induction (B, n = 6). Scale bar, 500 µm. Retinal trypsin digestion was performed to examine retinal acellular capillaries and representative images were captured with oil immersion with a 60 × objective lens. Arrow indicated acellular capillaries. Quantification was averaged from at least 10 randomly selected fields per retina (C, n = 6). Scale bar, 10 µm. ELISA assays were conducted to detect the expression of TNF‐α, VEGF, IL‐6, IL‐1β, and ICAM‐1 protein in retinal lysates (D, n = 4). *p < 0.05 versus Ctrl; ^#^ *p* < 0.05 between the marked groups. The significant difference was evaluated by one‐way ANOVA followed by post hoc Bonferroni test.

Microvascular dysfunction has been recognized as a hallmark of DR.^[^ ^5^ ^]^ We next investigate the role of 5′tiRNA‐His‐GTG in retinal vascular dysfunction induced by diabetes in vivo. Following 2 months of diabetes induction, the mice underwent intravitreal injections twice a month for an additional 2 months. The injections included negative control (NC) agomir, NC antagomir, 5′tiRNA‐His‐GTG agomir, or 5′tiRNA‐His‐GTG antagomir. Injection of 5′tiRNA‐His‐GTG agomir or antagomir had no discernible impact on blood glucose levels or body weight in diabetic mice, as shown in Table S1 (Supporting Information). The delivery efficiency of the 5′tiRNA‐His‐GTG agomir and antagomir was confirmed through qRT‐PCR assays, as shown in **Figure** **3**A. 5′tiRNA‐His‐GTG antagomir is an antisense oligonucleotide designed with a complementary sequence to 5′tiRNA‐His‐GTG, enabling functional inhibition without affecting its expression levels. This antagomir incorporates phosphorothioate and cholesterol modifications at the 3′ end, phosphorothioate modification at the 5′ end, and full‐chain methylation. These structural enhancements improve its stability, making it suitable for prolonged in vivo applications.

DR is characterized by retinal inflammation, capillary degeneration, neovascularization, and vascular leakage due to the breakdown of blood‐retinal barrier (BRB). To assess the extent of vascular leakage, Evans blue assays were conducted to measure BRB permeability. Injection of 5′tiRNA‐His‐GTG agomir exacerbated retinal vascular leakage, while 5′tiRNA‐His‐GTG antagomir induced less retinal vascular leakage compared to NC agomir group after 4 months of diabetes induction (Figure 3B).

Pericyte loss and subsequent disappearance of endothelial cells can lead to the formation of acellular capillaries at the early stage of DR. Retinal trypsin digestion demonstrated that intravitreal injection of 5′tiRNA‐His‐GTG agomir promoted capillary degeneration as shown by increased number of acellular capillaries. By contrast, injection of 5′tiRNA‐His‐GTG antagomir reduced the number of acellular capillaries induced by diabetes (Figure 3C). Chronic low‐grade inflammatory disease is another hallmark of DR. To determine the biological effects of 5′tiRNA‐His‐GTG on diabetes‐induced retinal inflammation, ELISA assays were performed to detect the expressions of pro‐inflammatory cytokines and angiogenic factors after injection of 5′tiRNA‐His‐GTG agomir or antagomir. Compared with diabetic mice, the retinas in the 5′tiRNA‐His‐GTG agomir group exhibited a significant increase in the expression of TNF‐α, VEGF, IL‐6, IL‐1β, and ICAM‐1. Notably, intravitreal injection of 5′tiRNA‐His‐GTG antagomir significantly reversed retinal inflammation responses (Figure 3D). Collectively, these results suggest that 5′tiRNA‐His‐GTG plays a critical role in diabetes‐induced retinal vascular dysfunction in vivo.

### 2.4 5′tiRNA‐His‐GTG Regulates Diabetes‐Induced Retinal Neuronal Dysfunction In Vivo

**Figure 4:**
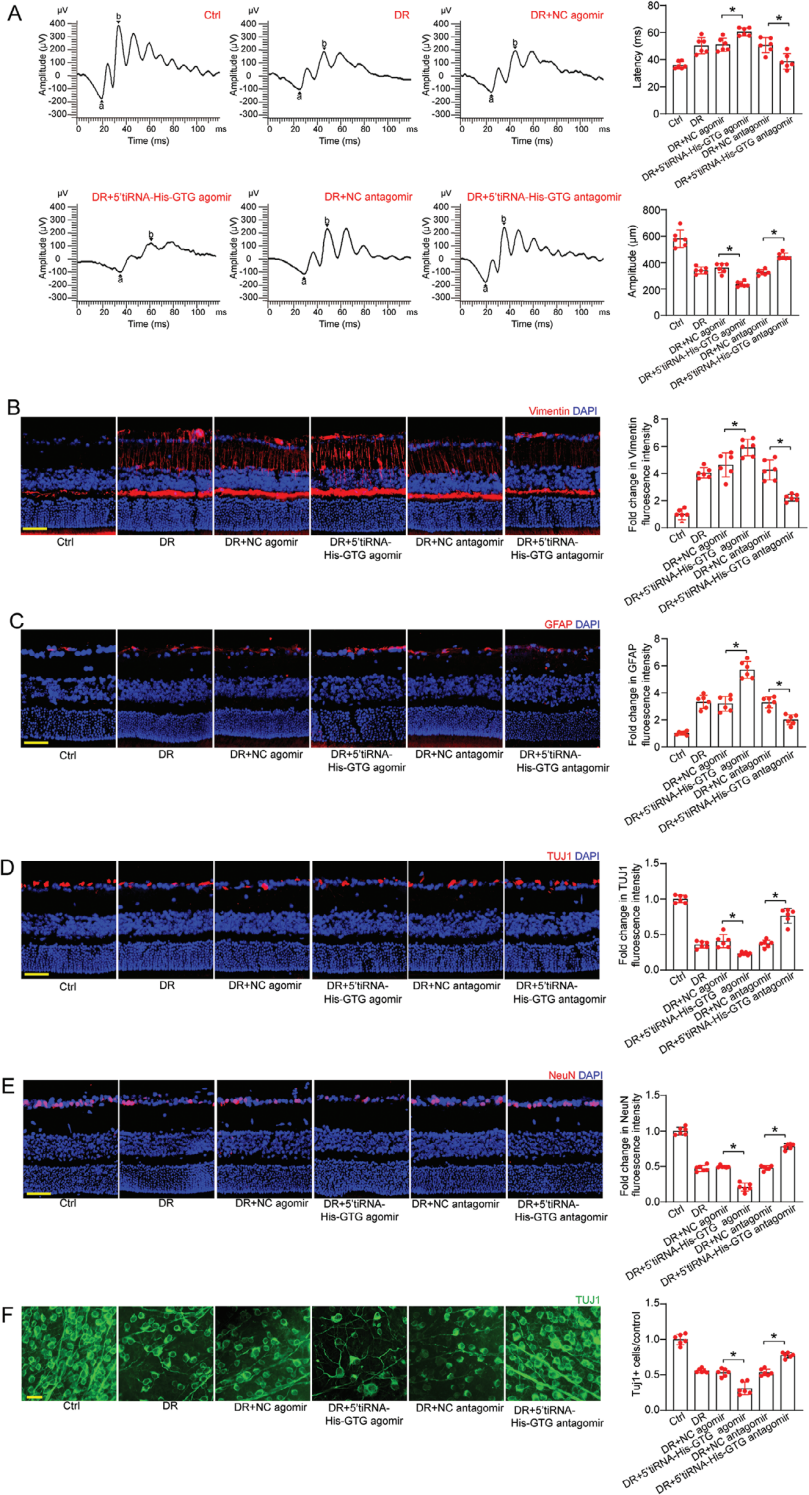
The effect of 5′tiRNA‐His‐GTG on diabetes‐induced retinal neuronal dysfunction in vivo. A) Flash Electroretinogram (fERG) was performed to detect visual function in non‐diabetic mice (Ctrl), STZ‐induced diabetic mice injected with negative control (NC) agomir, 5′tiRNA‐His‐GTG agomir, NC antagomir, and 5′tiRNA‐His‐GTG antagomir following 4‐month diabetes induction. Representative waves of six groups of mice measured through fERG were shown. Quantification of the amplitudes and latency of b‐wave (n = 6). B,C) Quantitative analyses of Vimentin (B) and GFAP (C) immunofluorescence intensity were conducted to detect retinal reactive gliosis. The representative images were shown (n = 6). Scale bar, 50 µm. D,E) Quantitative analyses of TUJ1 (D) and NeuN (E) immunofluorescence intensity were conducted to detect the survival of RGCs. The representative images were shown (n = 6). Scale bar, 50 µm. F) Retinal whole‐mounts were stained with TUJ1 to visualize RGCs. The number of surviving RGCs was compared with the control group to determine RGC survival (n = 6). Scale bar, 20 µm. *p < 0.05 versus Ctrl; ^#^ *p* < 0.05 between the marked groups. The significant difference was evaluated by one‐way ANOVA followed by post hoc Bonferroni test.

Neurodegeneration is recognized as another hallmark of DR, associated with microvascular injury and BRB breakdown.^[^ ^31^ ^]^ Given the effect of 5′tiRNA‐His‐GTG on Müller cell and neural cell function in vitro, we further investigated the role of 5′tiRNA‐His‐GTG in retinal neurodegeneration in vivo. Electroretinography (ERG) assays were conducted to evaluate visual function in diabetic mice 4 months after diabetes. The results revealed that, compared with DR group, up‐regulation of 5′tiRNA‐His‐GTG exacerbated neuronal dysfunction as shown by reduced amplitude of B‐waves and increased B‐wave latency. In contrast, down‐regulation of 5′tiRNA‐His‐GTG exhibited neuroprotective effects that could partially alleviate visual dysfunction induced by DR  (Figure **4**A).

Reactive gliosis and neural apoptosis are distinctive features of neurodegeneration.^[^ ^32^ ^]^ Immunostaining assays demonstrated that intravitreal injection of 5′tiRNA‐His‐GTG agomir significantly aggravated reactive gliosis as shown by increased vimentin and glial fibrillary acidic protein (GFAP) staining (Figure 4B,C), whereas intravitreal injection of 5′tiRNA‐His‐GTG antagomir alleviated gliosis reactivity in the retina. In addition, immunostaining with TUJ1 and NeuN revealed that the number of TUJ1‐ and NeuN‐ positive RGCs were decreased in 5′tiRNA‐His‐GTG agomir group (Figure 4D,E), whereas injection of 5′tiRNA‐His‐GTG antagomir could protect RGCs from injuries in DR (Figure 4B‐E). A similar result was observed in whole‐mounted retinas that were immunofluorescently stained with TUJ1 (Figure 4F).

Moreover, the expression of other retinal cell‐specific proteins, including calbindin (horizontal cells), calretinin (ganglion cells and amacrine cells), rhodopsin (rod and cone photoreceptor), and protein kinase Cα (PKCα; bipolar cells), were assessed in retinal slices. Immunostaining assays revealed that injection of 5′tiRNA‐His‐GTG agomir led to a reduction in calretinin‐labeled cells in the GCL layer, while the number of calretinin‐labeled cells in the GCL layer increased in 5′tiRNA‐His‐GTG antagomir group. However, there were no discernible changes in the number of calbindin‐labeled cells and calretinin‐labeled cells in the INL layer (Figure S4, Supporting Information). Rhodopsin and PKCα immunolabeling indicated that up‐regulation and down‐regulation of 5′tiRNA‐His‐GTG had no discernible effects on photoreceptors and bipolar cells (Figure S4, Supporting Information). Overall, these results suggest that 5′tiRNA‐His‐GTG regulates diabetes‐induced retinal neuronal dysfunction by affecting retinal gliosis and RGC survival.

### 2.5 5′tiRNA‐His‐GTG Regulates Diabetes‐Induced Visual Impairment In Vivo

**Figure 5:**
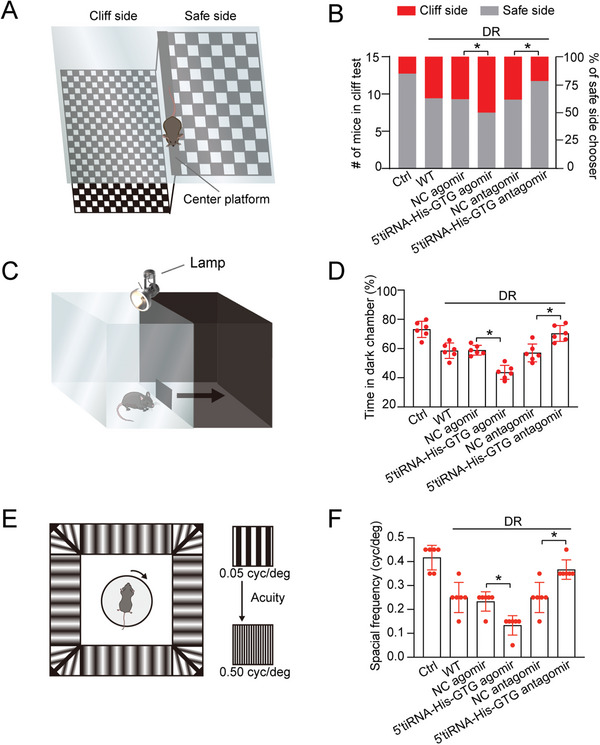
The effect of 5′tiRNA‐His‐GTG on diabetes‐induced visual impairment in vivo. A – F) Diabetic C57BL/6J mice received intravitreal injections of negative control (NC) agomir, 5′tiRNA‐His‐GTG agomir, NC antagomir, or 5′tiRNA‐His‐GTG antagomir or were left untreated (WT) twice a month following 4‐month diabetes induction. The non‐diabetic C57BL/6J mice were taken as the control (Ctrl) group. Visual depth perception was detected by visual cliff test. The schematic diagram is shown (A). The left y‐axis showed the number of mice stopping or not stopping at the cliff, and the right y‐axis showed the percentage (B, n = 6). Light perception was detected by light/dark preference test. The schematic diagram is shown (C). The statistical results were expressed as the percentage of time spent in the dark compartment (D, n = 6). Visual acuity was detected by optokinetic response test. The schematic diagram is shown (E). The statistical results displayed the highest spatial frequency threshold established per eye (F, n = 6). *p < 0.05 between the marked groups. The significant difference was evaluated by one‐way ANOVA followed by the post hoc Bonferroni test.

Impaired visual function resulting from retinal neurovascular dysfunction is often irreversible during the advanced stages of DR. Four months after injection of STZ, diabetic mice received intravitreal injections of NC agomir, 5′tiRNA‐His‐GTG agomir, NC antagomir, or 5′tiRNA‐His‐GTG antagomir. Various visually guided behavioral tests were conducted to determine the impact of 5′tiRNA‐His‐GTG on diabetes‐induced visual dysfunction. Visual cliff experiments were first carried out to test whether altered expression of 5′tiRNA‐His‐GTG could affect visual depth perception. Mice were put on a center platform above checkerboard pattern, and their movements toward either side were recorded (**Figure** **5**A). The results showed that diabetic mice exhibited an obvious deficit in depth discrimination. Intravitreal injection of 5′tiRNA‐His‐GTG agomir aggravated this deficit as shown by an increased tendency to approach cliff side, whereas 5′tiRNA‐His‐GTG antagomir could improve depth discrimination (Figure 5B), suggesting that down‐regulation of 5′tiRNA‐His‐GTG could restore visual sensitivity to elevation effects. Next, light/dark preference tests were conducted to evaluate light perception in diabetic mice after intravitreal injection of 5′tiRNA‐His‐GTG agomir or antagomir. Mice were placed in a bright area and allowed to leave freely to a darker environment (Figure 5C). 5′tiRNA‐His‐GTG agomir group spent a longer time moving to the darker chamber than DR group, whereas less time was spent in the 5′tiRNA‐His‐GTG antagomir group (Figure 5D), suggesting a restoration of light perception after the down‐regulation of 5′tiRNA‐His‐GTG antagomir. Moreover, visual acuity was estimated by optokinetic response tests. Mice were placed on a central elevated platform surrounded by display monitors with rotating visual stimuli, and their head movements were recorded (Figure 5E). Improved visual acuity was recorded following intravitreal injection of 5′tiRNA‐His‐GTG antagomir, with a higher frequency to track the systematically increased grating. Conversely, 5′tiRNA‐His‐GTG agomir group exhibited reduced visual acuity compared to DR group (Figure 5F), demonstrating that down‐regulation of 5′tiRNA‐His‐GTG could delay diabetes‐induced decrease in visual acuity. Collectively, these findings suggest the therapeutic potential of 5′tiRNA‐His‐GTG in mitigating visual impairment associated with DR.

### 2.6 5′tiRNA‐His‐GTG Regulates Neurovascular Dysfunction via Arachidonic Acid Metabolism and CYP Pathway

**Figure 6:**
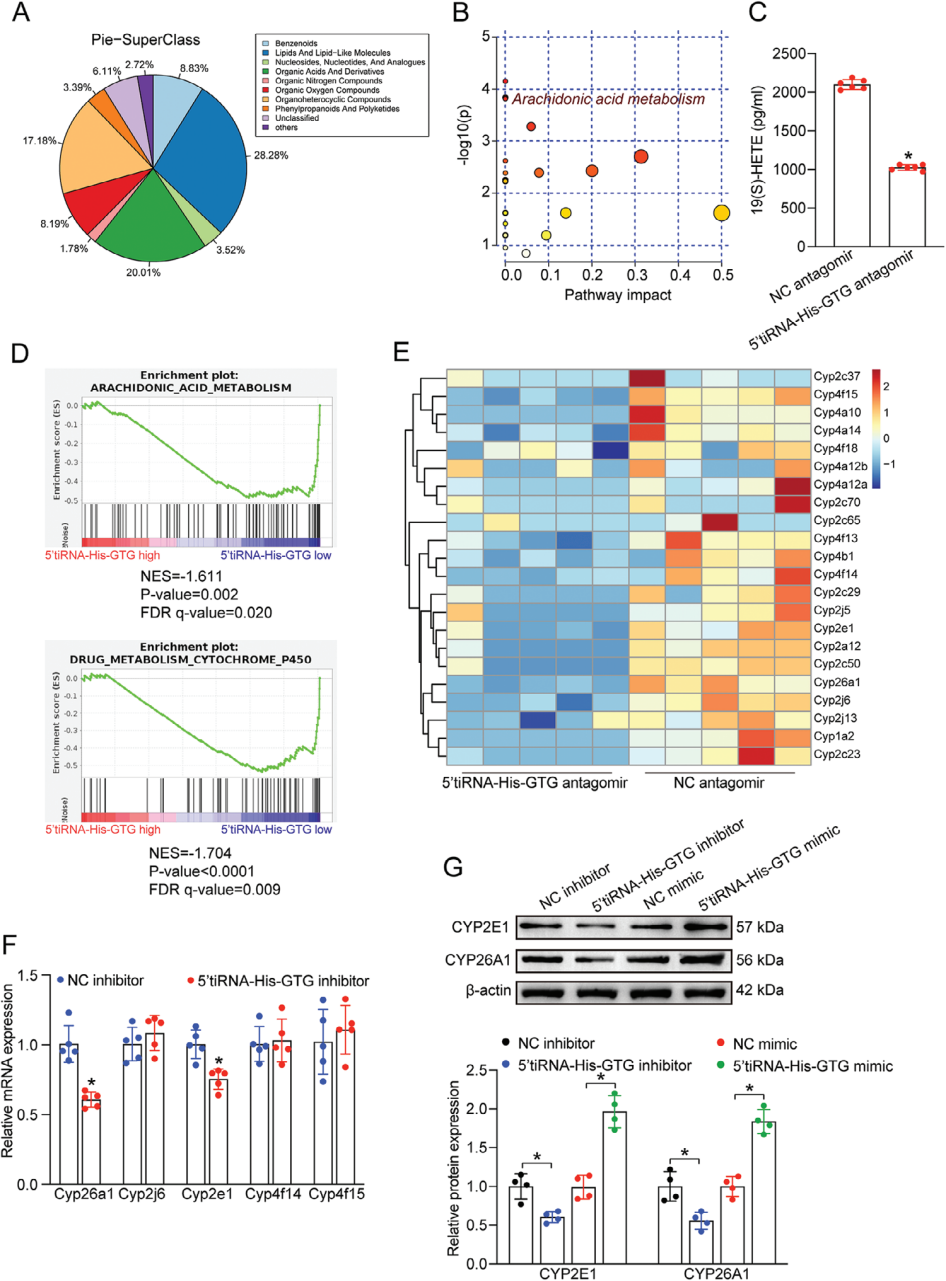
Metabolic and transcriptional changes associated with 5′tiRNA‐His‐GTG in regulating neurovascular dysfunction. A‐E) Diabetic C57BL/6J mice received intravitreal injections of negative control (NC) antagomir or 5′tiRNA‐His‐GTG antagomir twice a month following 4‐month diabetes induction, and then the retinas were collected for metabolomic and transcriptomic analyses. The pie chart displayed the differentially expressed metabolites between NC antagomir group and 5′tiRNA‐His‐GTG antagomir group (A). Pathway enrichment and topology analysis of differentially expressed metabolites. The size of the bubble indicates the pathway impact value. The vertical coordinate and color of the bubble indicate the *p* value (B). Retina 19(S)‐HETE metabolites were measured by ELISAs (C, n = 6, *p < 0.05, Student's *t‐*test). GSEA pathway analysis of NC antagomir group versus 5′tiRNA‐His‐GTG antagomir group for AA metabolism, or drug metabolism CYP pathways (D, n = 5). Differential gene expression heatmap of top‐altered CYP enzymes genes between diabetic mice injected with 5′tiRNA‐His‐GTG antagomir and NC antagomir (E, n = 5). F) The levels of CYP26A1, CYP2J6, CYP2E1, CYP4F14, and CYP4F15 expression were detected by qRT‐PCRs in Müller cells following transfection of NC inhibitor or 5′tiRNA‐His‐GTG inhibitor (n = 5, *p<0.05 versus NC inhibitor group, Student's *t‐*test). G) Western blots and quantitative analysis were performed to examine the protein levels of CYP2E1 and CYP26A1 in Müller cells following transfection of 5′tiRNA‐His‐GTG mimic, NC mimic, 5′tiRNA‐His‐GTG inhibitor, or NC inhibitor (n = 4, *p < 0.05 between the marked groups, one‐way ANOVA followed by the post hoc Bonferroni test).

We then explored the potential mechanism by which 5′tiRNA‐His‐GTG regulates retinal neurovascular dysfunction in diabetic condition. Metabolomic and transcriptomic analyses were conducted in diabetic mice after intravitreal injection of 5′tiRNA‐His‐GTG antagomir or NC antagomir at 4 months after diabetes induction. Metabolomic analysis revealed that lipids and lipid‐like molecules were mainly involved in the altered metabolite (**Figure** **6**A). KEGG analysis was performed to understand the metabolic pathways associated with the change of retinal metabolic profiles following injection of 5′tiRNA‐His‐GTG antagomir. The results revealed great enrichment in arachidonic acid (AA) metabolism pathway (Figure 6B). Moreover, ELISA assays confirmed that the content of differentially expressed metabolite 19(S)‐HETE was markedly reduced in the 5′tiRNA‐His‐GTG antagomir group (Figure 6C). For transcriptomics analysis, gene set enrichment analysis (GSEA) demonstrated that 5′tiRNA‐His‐GTG knockdown induced a marked reduction of pathways involved in AA metabolism and drug metabolism cytochrome P450 (CYP) pathways (Figure 6D). AA is mainly metabolized by CYPs, cyclooxygenase (COX), and lipoxygenase (LOX) pathways, while 19(S)‐HETE is metabolized through CYP pathway.^[^ ^33^, ^34^ ^]^ In addition, the heat map of transcriptomic analyses also demonstrated decreased mRNA expression of CYP enzymes in the 5′tiRNA‐His‐GTG antagomir group (Figure 6E). The transcriptomic profiling was consistent with the above metabolomic results and further revealed reduced levels of 19(S)‐HETE metabolites, indicating that 5′tiRNA‐His‐GTG positively regulates AA metabolism through CYP pathway. Furthermore, 5 differentially expressed genes with the most significant differences were selected for validation in Müller cells. qRT‐PCR assays demonstrated that transfection of 5′tiRNA‐His‐GTG inhibitor caused a marked reduction of CYP26A1 and CYP2E1 mRNA expression but had no effects on the expression of CYP2J6, CYP4F14, and CYP4F15 (Figure 6F). Western blot analysis also revealed that the expression levels of CYP2E1 and CYP26A1 increased following transfection of 5′tiRNA‐His‐GTG mimic. By contrast, transfection of 5′tiRNA‐His‐GTG inhibitor reduced the expression levels of CYP2E1 and CYP26A1 (Figure 6G). It is important to mention that CYP2E1, as a monooxygenase, metabolizes AAs to HETEs and plays a crucial role in AA metabolism, while CYP26A1 primarily participates in retinoic acid metabolism.^[^ ^35^, ^36^ ^]^ Collectively, these findings suggest that 5′iRNA‐His‐GTG regulates neurovascular dysfunction by activating CYP2E1‐19(S)‐HETE in AA metabolism.

### 2.7 Regulation of Retinal Neurovascular Dysfunction by 5′tiRNA‐His‐GTG‐CYP2E1‐19(S)‐HETE Signaling Axis

**Figure 7:**
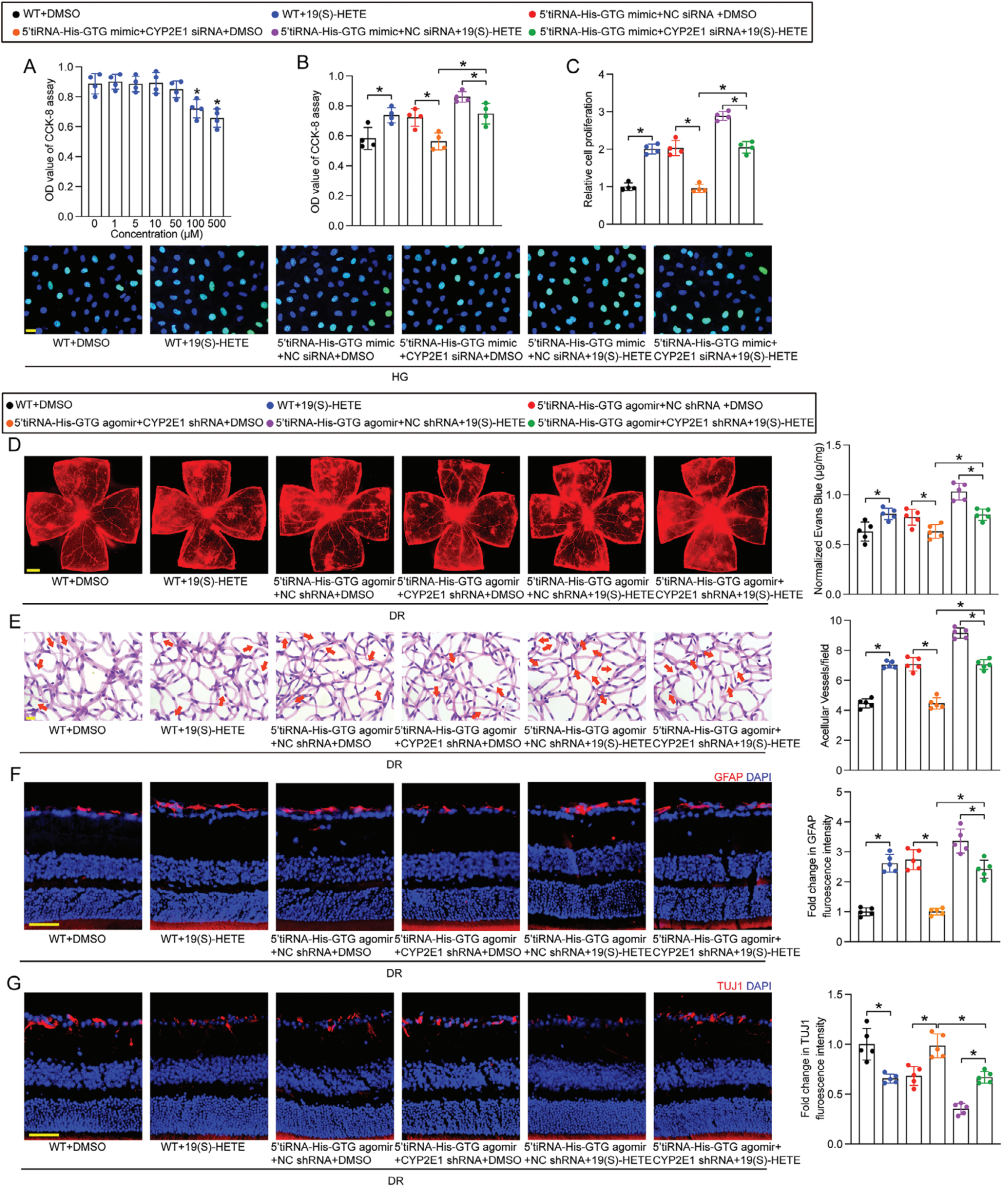
5′tiRNA‐His‐GTG‐CYP2E1‐19(S)‐HETE signaling axis regulates retinal neurovascular dysfunction. A) Müller cells were cultured with different concentrations of 19(S)‐HETE (1‐500 µM) or left untreated (0 µM, Ctrl) for 24 h. Cell viability was detected by CCK‐8 assays (n = 4; * *p*< 0.05 versus Ctrl, one‐way ANOVA followed by the post hoc Bonferroni test). B,C) Müller cells were transfected with negative control (NC) mimic (WT), 5′tiRNA‐His‐GTG mimic plus NC siRNA, or 5′tiRNA‐His‐GTG mimic plus CYP2E1 siRNA, and then treated with 19(S)‐HETE (10 µM) or DMSO for 24 h. Cell viability was examined by CCK‐8 assays (B, n = 4). EdU staining and quantitative analysis were performed to evaluate cell proliferation. EdU, green; DAPI, blue. Scale bar, 20 µm (C, n = 4). D – G) Diabetic C57BL/6J mice received intravitreal injections of NC agomir (WT), 5′tiRNA‐His‐GTG agomir plus NC shRNA, or 5′tiRNA‐His‐GTG agomir plus CYP2E1 shRNA for 2 months, and then treated with 19(S)‐HETE (50 µM) or DMSO. Retinal vasopermeability (D, scale bar, 500 µm), acellular capillaries (E, scale bar, 10 µm), reactive gliosis (F, scale bar, 50 µm), and RGC degeneration (G, scale bar, 50 µm) were detected to evaluate the role of 5′tiRNA‐His‐GTG‐CYP2E1‐19(S)‐HETE signaling axis in retinal neurovascular dysfunction in vivo (n = 5).

To determine the non‐toxic concentration of 19(S)‐HETE in Müller cells, a range from 1 to 500 µM was administered for 24 h. CCK‐8 assay demonstrated that the viability of Müller cells remained unaffected within the concentration range of 1 µM to 50 µM (**Figure** **7**A). Accordingly, 10 µM was selected for the subsequent experiment. We then assessed whether CYP2E1‐mediated AA metabolism played a role in the biological effects of 5′tiRNA‐His‐GTG.

CCK‐8 and EdU assays revealed that treatment with 10 µM of 19(S)‐HETE for 24 h enhanced the viability and proliferation of Müller cells, akin to the effects observed with 5′tiRNA‐His‐GTG mimic transfection. However, CYP2E1 silencing reversed the impacts of 5′tiRNA‐His‐GTG mimic on Müller cell function. Additionally, 19(S)‐HETE treatment partially counteracted the decrease of cell viability and proliferation resulting from CYP2E1 knockdown in 5′tiRNA‐His‐GTG‐overexpressing Müller cells (Figure 7B,C).

Evans blue and PAS assays were then carried out to investigate the effect of 5′tiRNA‐His‐GTG‐CYP2E1‐19(S)‐HETE signaling on retinal vascular dysfunction in vivo. The results showed that 19(S)‐HETE treatment aggravated retinal vascular dysfunction induced by diabetes, similar to the effects of injecting 5′tiRNA‐His‐GTG agomir. However, CYP2E1 silencing reversed the impacts of 5′tiRNA‐His‐GTG agomir on retinal vascular dysfunction. Additionally, 19(S)‐HETE treatment partially exacerbated reduced vascular dysfunction due to CYP2E1 knockdown in 5′tiRNA‐His‐GTG‐upregulated diabetic mice (Figure 7D,E).

The role of 5′tiRNA‐His‐GTG‐CYP2E1‐19(S)‐HETE signaling axis in retinal neurodegeneration was also investigated in vivo. Immunostaining assays revealed that exogenous 19(S)‐HETE administration aggravated retinal neurodegeneration, characterized by increased reactive gliosis and RGC degeneration in STZ‐induced diabetic mice, similar to the effects of injection of 5′tiRNA‐His‐GTG agomir. Conversely, CYP2E1 silencing reversed the impact of 5′tiRNA‐His‐GTG agomir on retinal neurodegeneration. Moreover, 19(S)‐HETE treatment partially counteracted the protective effects on retinal neurodegeneration resulting from CYP2E1 knockdown in 5′tiRNA‐His‐GTG‐upregulated diabetic mice (Figure 7F,G). Collectively, these findings indicate the involvement of 5′tiRNA‐His‐GTG‐CYP2E1‐19(S)‐HETE signaling axis in retinal neurovascular dysfunction.

## 3 Discussion

DM is a multifaceted metabolic disorder arising from insulin resistance.^[^ ^37^ ^]^ DR, a severe complication of DM, causes impairment of the entire retinal neurovascular unit, encompassing retinal vascular dysfunction, glial activation, and neural apoptosis.^[^ ^25^ ^]^ However, the precise mechanisms underlying DR‐induced neurovascular dysfunction remain elusive. Here, we clarify the significance of a tRNA‐derived stress‐induced small RNA, 5′tiRNA‐His‐GTG, which is up‐regulated during the progression of DR. Up‐regulation of 5′tiRNA‐His‐GTG accelerates retinal vascular dysfunction, exacerbates retinal neurodegeneration, and impairs visual function and visually‐guided behaviors in diabetic mice. In contrast, inhibition of 5′tiRNA‐His‐GTG exhibits neurovascular protective effects. Mechanistically, down‐regulation of 5′tiRNA‐His‐GTG causes a marked reduction of AA metabolism‐modulated cytochrome P450 (CYP) pathway by decreasing CYP2E1 expression. Furthermore, the 5′tiRNA‐His‐GTG‐CYP2E1‐19(S)‐HETE signaling axis is implicated in retinal neurovascular dysfunction. Collectively, this study suggests that down‐regulation of 5′tiRNA‐His‐GTG holds therapeutic promise in rescuing visual function by neurovascular protection.

Transfer RNA (tRNA)‐derived small RNAs (tsRNAs), an emerging class of functional non‐coding RNAs, play important roles in gene expression, protein translation, and stress responses. Recent studies have identified the dysregulation of tsRNAs in various diseases, such as metabolic disorders, cancer, inflammation, and neurological ailments. Persistent hyperglycemia results in retinal damage through oxidative stress and hypoxia, prompting the transcription of ANG, a member of the RNase A family. Specifically, transfer RNA halves (tiRNAs), a subtype of tsRNAs, are generated when mature tRNAs undergo cleavage at the anti‐codon loop under stress condition.^[^ ^38^ ^]^ In this study, we reveal that ANG is up‐regulated response to high glucose stress. We further establish that the expression level of 5′tiRNA‐His‐GTG is reduced upon ANG knockdown but not Dicer knockdown, indicating that the production of 5′tiRNA‐His‐GTG is induced by ANG‐mediated anticodon loop cleavage. In both in vitro and in vivo studies, 5′tiRNA‐His‐GTG exhibits up‐regulation in HG‐treated Müller cells, diabetic retinas, and clinical samples from patients with DR. Elevated levels of 5′tiRNA‐His‐GTG triggers retinal vascular dysfunction and neurodegeneration, contributing to retinal glial activation, neuronal apoptosis, acellular capillary formation, and vascular leakage. Consequently, down‐regulation of 5′tiRNA‐His‐GTG emerges as a potential strategy to manage vasculopathy and neuropathy during DR.

As the extension of the brain, the retina shares pathological mechanisms with central nervous system (CNS) diseases due to their common embryonic origin, cell types, and signaling pathway.^[^ ^39^ ^]^ Growing evidence indicates that alterations in the inner retina often manifest at earlier stages than in the CNS during neurodegenerative diseases.^[^ ^40^, ^41^ ^]^ The retina acts as a unique “window” for non‐invasive identification and monitoring of changes associated with CNS diseases. Moreover, retinal NVU closely parallels cerebral NVU, comprising neurons, glial cells, and vascular components.^[^ ^42^ ^]^ Chronic hyperglycemia can cause cell injuries within retinal NVU through oxidative stress, hypoxia, endoplasmic reticulum stress, and inflammation, culminating in NVU dysfunction.^[^ ^27^ ^]^ NVU impairment is a pivotal event in the pathogensis of DR, causing the disruption of inner blood‐retinal barrier, contributing to vision loss in DR patients.^[^ ^43^ ^]^ Components of retinal NVU intricately interact to regulate local blood supply, ensuring optimal oxygen and nutrient delivery to match neuronal energy demand.^[^ ^44^ ^]^ Prolonged diabetes adversely affects various components of retinal NVU, with glial‐mediated neurovascular coupling being fundamental for maintaining the homeostasis of retinal microenvironment.^[^ ^45^ ^]^ Müller cells play a crucial role in providing structural and metabolic support to neurons, as well as regulating blood flow in retinal vasculature.^[^ ^46^ ^]^ The expression of 5′tiRNA‐His‐GTG is up‐regulated in Müller cells under high glucose stress, underscoring their responsiveness to 5′tiRNA‐His‐GTG in diabetic condition. Co‐culturing with Müller cells amplifies angiogenic effects on ECs and exacerbates high glucose‐induced neuronal injury as shown by enhanced proliferation, migration, and tube formation ability of ECs, coupled with reduced viability and increased apoptosis of RGCs. Müller cells exhibit pro‐angiogenic effects on endothelial function and deleterious effects on RGC function in diabetes. Notably, down‐regulation of 5′tiRNA‐His‐GTG in Müller cells significantly attenuates angiogenic effects in ECs and enhances protective effects on RGCs.

In diabetes, Müller cells primarily undergo reactive gliosis, a process contributing to the production of inflammatory mediators and the release of various signaling molecules affecting the function of neighboring blood vessels and neurons.^[^ ^47^, ^48^ ^]^ We show that intravitreal injection of 5′tiRNA‐His‐GTG antagomir mitigates vascular leakage, capillary degeneration, and retinal inflammation in diabetic mice. Moreover, down‐regulation of 5′tiRNA‐His‐GTG ameliorates retinal reactive gliosis and enhances the survival of RGCs, as evidenced by reduced GFAP and Vimentin staining, along with increased TUJI and NeuN staining. These findings align with the impact of 5′tiRNA‐His‐GTG silencing on visually guided behavior mediated by DR. Therefore, reducing the expression of 5′tiRNA‐His‐GTG has the potential to delay diabetes‐induced complications by preserving retinal microenvironment homeostasis and restoring the integrity of NVU.

Due to the retina is a highly metabolically active tissue,^[^ ^21^ ^]^ this prompted us to conduct metabolomic and transcriptomic analyses to elucidate alterations in metabolic pathways, metabolites, and key genes associated with improved neurovascular function observed upon 5′tiRNA‐His‐GTG knockdown in diabetic mice. The multi‐omic data analysis shows that 5′tiRNA‐His‐GTG regulates neurovascular dysfunction through AA metabolism‐modulated CYP pathway. Abnormal AA metabolism is known to contribute to endothelial dysfunction, inflammation, and atherosclerosis^.[^ ^49^ ^]^ and CYP pathway serves as the third pathway for AA metabolism.^[^ ^50^ ^]^ In this study, 5′tiRNA‐His‐GTG knockdown leads to the downregulation of AA metabolism and drug metabolism CYP pathways. Specifically, CYP2E1 expression is suppressed in Müller cells following the transfection with 5′tiRNA‐His‐GTG inhibitor. Elevated CYP2E1 expression has been linked not only to liver damage from oxidative stress but also to toxicity in other organs.^[^ ^51^ ^]^ Induction of CYP2E1 has been reported to contribute to increased lipid peroxidation and apoptosis, leading to heightened permeability of blood‐brain barrier and neurodegeneration.^[^ ^52^ ^]^ Knockdown of CYP2E1 can partially interrupt the effects of 5′tiRNA‐His‐GTG on retinal vascular leakage, acellular capillaries, reactive gliosis, and RGC survival, thereby restoring the function of NVU.

In response to metabolic demands of the retina, both neurons and glial cells can release AA metabolites to alter blood flow during neural activity and energy production responses.^[^ ^53^ ^]^ AA undergoes specific metabolism by cytochrome P450 enzymes to generate vasoactive products, including epoxyeicosatrienoic acids (EETs) and hydroxyeicosatetraenoic acids (HETEs).^[^ ^54^ ^]^ Previous studies have shown that CYP2E1 produces two main lipid mediators, 18‐HETE and 19‐HETE. 19‐HETE constitutes a significant proportion of total metabolites, with 70% being 19(S)‐HETE and the remaining 30% being 19(R)‐HETE.^[^ ^55^ ^]^ 19(S)‐HETE acts as an inflammatory mediator and plays a crucial role in the onset and progression of cardiovascular disease.^[^ ^56^ ^]^ In this study, the level of 19(S)‐HETE is markedly reduced in 5′tiRNA‐His‐GTG antagomir group. Treatment of 19(S)‐HETE mimics the detrimental effects of up‐regulation of 5′tiRNA‐His‐GTG on neurovascular dysfunction. 19(S)‐HETE treatment also partially counters the protective effects on retinal neurodegeneration and vascular dysfunction due to CYP2E1 knockdown in 5′tiRNA‐His‐GTG‐upregulated diabetic mice. Hence, it is not surprising that 5′tiRNA‐His‐GTG‐CYP2E1‐19(S)‐HETE axis is involved in retinal neurovascular dysfunction.

However, the present study has some limitations. The ARPE‐19 cells used to assess the expression pattern of 5′tiRNA‐His‐GTG in various retinal cells do not fully replicate the physiology and pathology of normal primary cells, which may affect the generalizability of the findings. Future research should employ more physiologically relevant models to validate these results. Additionally, while our study focused on Müller cells, RGCs, and ECs within NVU, the role of other retinal cell types, such as microglia, remains to be fully elucidated. Microglia‐mediated neovascularization is particularly relevant in the pathophysiology of DR due to hypoxic‐induced neovascularization. Microglia can infiltrate the basement membrane of the inner blood‐retinal barrier and phagocytize endothelial cells, potentially leading to iBRB breakdown and vascular impairment.^[^ ^57^ ^]^ Future studies should investigate the impact of microglial activation on the mechanism of 5′tiRNA‐His‐GTG and explore their potential contributions to neurovascular dysfunction in DR.

In conclusion, our study provides compelling evidence elucidating the involvement of 5′tiRNA‐His‐GTG in NVU dysfunction in diabetic mice. Additionally, 5′tiRNA‐His‐GTG regulates DR through AA metabolism regulated by CYP2E1. Diabetic mice deficient in 5′tiRNA‐His‐GTG exhibites notable improvement in visual function and visually guided behavior, underscoring the potential of targeting 5′tiRNA‐His‐GTG as a promising therapeutic strategy for treating diabetic vasculopathy and neuropathy. These findings provide strong evidence supporting the development of targeted therapies for DR treatment.

## 4 Experimental Section

### Clinical Sample

The clinical samples were collected using a standard sterilization procedure at the Affiliated Eye Hospital of Nanjing Medical University. At the beginning of the surgery, a total of 50 µL aqueous humor samples were collected from each patient with trauma, cataracts, or DR. Retinal membranes were obtained from patients with proliferative DR (n = 10) or idiopathic epiretinal membrane (n = 10) undergoing pars plana vitrectomy. The relevant information about the patients is shown in Tables S2, S3 (Supporting Information).

### Induction of Diabetic Mice

Male C57BL/6J mice aged at 8 weeks were obtained from the Animal Core Facility of Nanjing Medical University (Nanjing, Jiangsu, China). Diabetes was induced in C57BL/6 mice by intraperitoneal injection of streptozotocin (STZ, 50 mg kg^−1^, Biofroxx) for 5 consecutive days. Similar injections of citrate buffer were administered to age‐matched animals serving as controls. Diabetes was confirmed by elevated blood glucose level in tail vein (≥16.7 mmol l^−1^) after one week of the last STZ injection. The mice were housed in a controlled environment (lighting, 12‐h light/dark cycle; temperature, 25 ± 2 °C; humidity, 50 ± 10%).

### Cell Transfection

5′tiRNA‐His‐GTG mimic, 5′tiRNA‐His‐GTG inhibitor, negative control (NC) mimic, and NC inhibitor labeled by fluorescein amidite (FAM) were purchased from RiboBio Co., Ltd. (Guangzhou, China). Müller cells were seeded into plates one day prior to transfection. Upon reaching 85% confluency, Müller cells were transfected using Lipofectamine 2000 reagent (Invitrogen) following the manufacturer's protocol. The final concentrations of mimics and inhibitors were 50 nM. After 12 h, the medium was changed, and the cells were cultured at 37 °C for another 36 h in preparation for further research. In addition, transfection efficiency was evaluated under a fluorescence microscope at 24 h. RNA oligonucleotide sequences were shown in Table S4 (Supporting Information).

### Cell Co‐Culture Assay

Cell co‐culture system was used to explore the crosstalk between Müller cells and HRMECs or between Müller cells and RGCs. HRMECs or RGCs were seeded at the bottom chamber and Müller cells were simultaneously seeded into the upper chamber in a transwell culture system (0.4‐µm pore size; Millipore, PTHT24H48). HRMECs (2 × 10^5^ cells per well) or RGCs (2.5 × 10^5^ cells per well) were seeded in 24‐well plates with 500 µL of specific cell medium. After these cells adhered for 6–8 h, the medium was changed into 1 mL of DMEM/F12 with 10% FBS. Then, Müller cells (1 × 10^5^ cells per well) transfected with negative control (NC) mimic, 5′tiRNA‐His‐GTG mimic, NC inhibitor, 5′tiRNA‐His‐GTG inhibitor, or left untreated were cultured on the upper chamber and co‐cultured with HRMECs or RGCs at 37 °C for additional 24 h or 48 h.

### 5‐Ethynyl‐2′‐Deoxyuridine (EdU) Detection

Cell proliferation was detected according to the instruction of the BeyoClick EdU kit with Alexa Fluor 488 (Beyotime, C0071S). Treated cells were incubated at 37 °C for 3 h with a final concentration of 10 µM EdU reagent prior to 4% paraformaldehyde (PFA) fixation for 15 min. Then, 1 mL of permeable solution was added at room temperature for 15 min, followed by incubation with the click reaction solution in the dark for 30 min. Hoechst 33 342 was used to stain nucleic acids. The number of EdU‐positive cells was counted under a fluorescent microscope (Olympus, USA) in five random fields. ImageJ was used to calculate cell proliferation. Flow cytometry was performed on Beckman Coulter CytoFLEX LX, and the data were analyzed using FlowJo software (Tree Star, United States).

### Evans Blue Dye Perfusion

To quantify the permeability of retinal vasculature, mice were anesthetized and received a femoral vein injection of 45 mg kg^−1^ Evans blue dye in PBS (sterilized by passing through a 0.22 m filter), and the blood samples were collected. Then, PBS was perfused into the ascending aorta of mice to remove Evans blue dye from blood vessels. The eyeballs were fixed in 4% PFA for 30 min at room temperature. Both eyeballs were enucleated and cut in half along the equator. One retina from each mouse was removed freshly by dissection and split into four pieces for immunofluorescence observation using a fluorescence microscope (Olympus IX 73 DP80, Japan). The other retina was dissected for extracting Evans blue dye. The retinas were dried at 78 °C for 5 h and then weighed. Formamide (200 µL) was added to each retina and incubated at 78 °C overnight. Following dye extraction, the resultant mixtures were centrifuged at 12 000 *g* for 45 min. Each sample was measured at 620 nm and 740 nm (background) using a spectrophotometer. Blood samples were treated similarly. The quantity of Evans Blue dye in formamide was determined using a standard curve with the following formula:

(1) $$\frac{\mathrm{Retinal}\;\mathrm{Evans}\;\mathrm{blue}\;\mathrm{concentration}\left(\mathrm{mg}/\mathrm{ml}\right)/\;\mathrm{Retinal}\;\mathrm{weight}\left(\mathrm{mg}\right)}{\mathrm{Blood}\;\mathrm{Evans}\;\mathrm{blue}\;\mathrm{concentration}\left(\mathrm{mg}/\mathrm{ml}\right)\;\times \;\mathrm{Circulation}\;\mathrm{time}\left(h\right)}$$

### Retinal Trypsin Digestion

Eyeballs were enucleated and fixed in 10% neutral formaldehyde for 24 h. The retinas were rinsed in distilled water at least 4–5 times for 30 min, and digested with 3% Trypsin (1:250, BioFroxx, 1004GR025) in 0.1 M Tris buffer (pH7.8) at 37 °C for 1 h to isolate retinal vasculature. The air‐dried samples were stained with periodic acid‐Schiff and hematoxylin to highlight basement membranes and capillary nuclei. Each retina had at least 10 randomly selected fields (600 × magnification). The number of acellular capillaries was counted in each field using an Olympus IX‐73 microscopy.

### Electroretinogram (ERG)

The mice were dark‐adapted overnight and subsequently anesthetized under dim red light to maintain darkness. 1% tropicamide was applied topically to dilate the pupils of the mice. Then, topical use of 1% carboxymethylcellulose was applied to the cornea to improve corneal contact and avoid dryness. The recording electrode made of a gold wire loop was placed on the central cornea. As reference electrodes, two 25‐gauge platinum needles were placed around the eye. The ground electrode was attached to the tail. In the presence of increasing light stimulus, photopic flash reactions were assessed after a scotopic intensity series was recorded. The B‐wave amplitude was measured from A‐wave trough to B‐wave peak. Additionally, B‐wave latency was acquired. The Roland Consult Color Ganzfeld Q450C recording equipment was used for stimulus presentation and data collection. During the experiment, the mice were put on a warming bed to maintain body temperature.

### Visual Cliff Test

The apparatus consisted of two clear plexiglass boxes (40 cm × 20 cm × 50 cm), blake‐and‐white checkered paper (checker size: 2.5 cm × 2.5 cm), a center platform (40 cm × 4 cm × 4 cm), and a clear plexiglass floor. Two clear plexiglass boxes were placed upside down and side by side. The clear plexiglass floor was elevated 50 cm off the ground A shallow side and a deep side, with the same checkered pattern are separated by a center platform to create the illusion of depth. Mice were placed on the center platform to freely roam in a square. Each mouse was given 10 tests for a 5‐min trial. The entire process was recorded with a video camera. The frequency of side choices was recorded and converted into a percentage to represent selection preference.

### Light/Dark Preference Test

A black box (30 × 20 × 30 cm) was inserted into a clear rectangular plexiglass box (30 × 40 × 30 cm) to separate into a bright zone and a dark zone with equal area. The dark box contained a small opening (5 cm × 5 cm) for free passage. The light chamber was illuminated by a 1000‐lux desk lamp, whereas the illumination in the dark chamber was 5 lux. After environment adaption, the experimental mouse was placed in the light/dark box with its head facing the dark chamber. The time spent in the light and dark areas was recorded for the 5 min test process.

### Optokinetic Response

Visual acuity was detected through a virtual optokinetic system (OptoMotry; CerebralMechanics Inc). Each mouse was placed on a central elevated platform surrounded by four display monitors with sinusoidal gratings rotating at 12° s^−1^. When the grating began at 100% contrast with spatial frequency start from 0.05 to 0.50 cycles deg^−1^, the mouse followed with a drifting scene until head turning was no longer observed, and the spatial frequency threshold was recorded. The entire experiment was monitored with a video camera, which was consistently adjusted to focus on the animal's head. The visual acuities reported for each eye represent the averages of six trials conducted on separate days.

### Statistics

The data was analyzed using GraphPad Prism 8 (GraphPad Software, San Diego, CA). The values are shown as mean ± SEM. For normally distributed data with equal variance, the significant difference was established using Student's *t*‐test when comparing two groups or one‐way or two‐way ANOVA followed by post hoc Bonferroni test when comparing more than two groups. For the non‐normally distributed data or data with uneven variances, the significance difference was established using the non‐parametric Mann‐Whitney's *U*‐test when comparing two groups or Kruskal‐Wallis's test followed by post‐hoc Bonferroni's test when comparing more than two groups. *p* < 0.05 was considered statistically significant.

### Study Approval

The in vivo experiments were conducted following the ARVO Statement for the Use of Animals in Ophthalmic and Vision Research and approved by the Experimental Animal Ethics Committee of the authors’ institute. All patients enrolled in this study provided informed consent in accordance with the Declaration of Helsinki, and the usage of patient samples was approved by the Ethical Committee of the authors’ institute.

## Conflict of Interest

The authors declare no conflict of interest.

## Author Contributions

J.Y., W.Y., J.‐Y.Z., Y.L., and J.‐H.L. contributed equally to this work. B.Y. and J.Y. designed the study. J.Y.Z., W.Y., Y.L., J.H.L., Y.K.J., and X.S.N. conducted the experiments and acquired data. J.Y.Z. and W.Y. analyzed the results and prepared figures. J.Y.Z., W.Y., and B.Y. wrote the manuscript. Y.L., J.H.L., Y.K.J., W.M., and X.S.N. developed the methods and data analysis. B.Y. and J.Y. contributed to the critical discussion of results. All authors critically reviewed and approved the final manuscript.

## Supporting information



Supporting Information

## Data Availability

The data that support the findings of this study are available from the corresponding author upon reasonable request.
