# Supplementary material for: Targeting tRNA‐Derived Non‐Coding RNA Alleviates Diabetes‐Induced Visual Impairment through Protecting Retinal Neurovascular Unit
Source: Adv Sci (Weinh). 2024 Nov 8;12(1):2411042. doi: 10.1002/advs.202411042 (PMC11714213; doi:10.1002/advs.202411042)
Supplement: Supplementary file 1 — Supporting Information [file ADVS-12-2411042-s001.docx]

**Supplemental data**

**
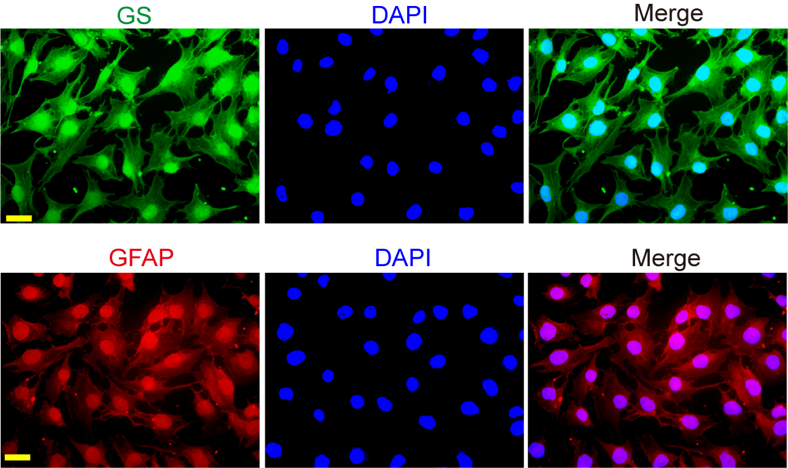
**

**Figure S1. Identification and purity detection of primary isolated Müller cells**

Immunofluorescent stainings of GS and GFAP were performed to label Müller cells. DAPI staining was used to label cell nuclei. Green, GS; Red, GFAP; Blue, DAPI. Scale bar, 20 μm.


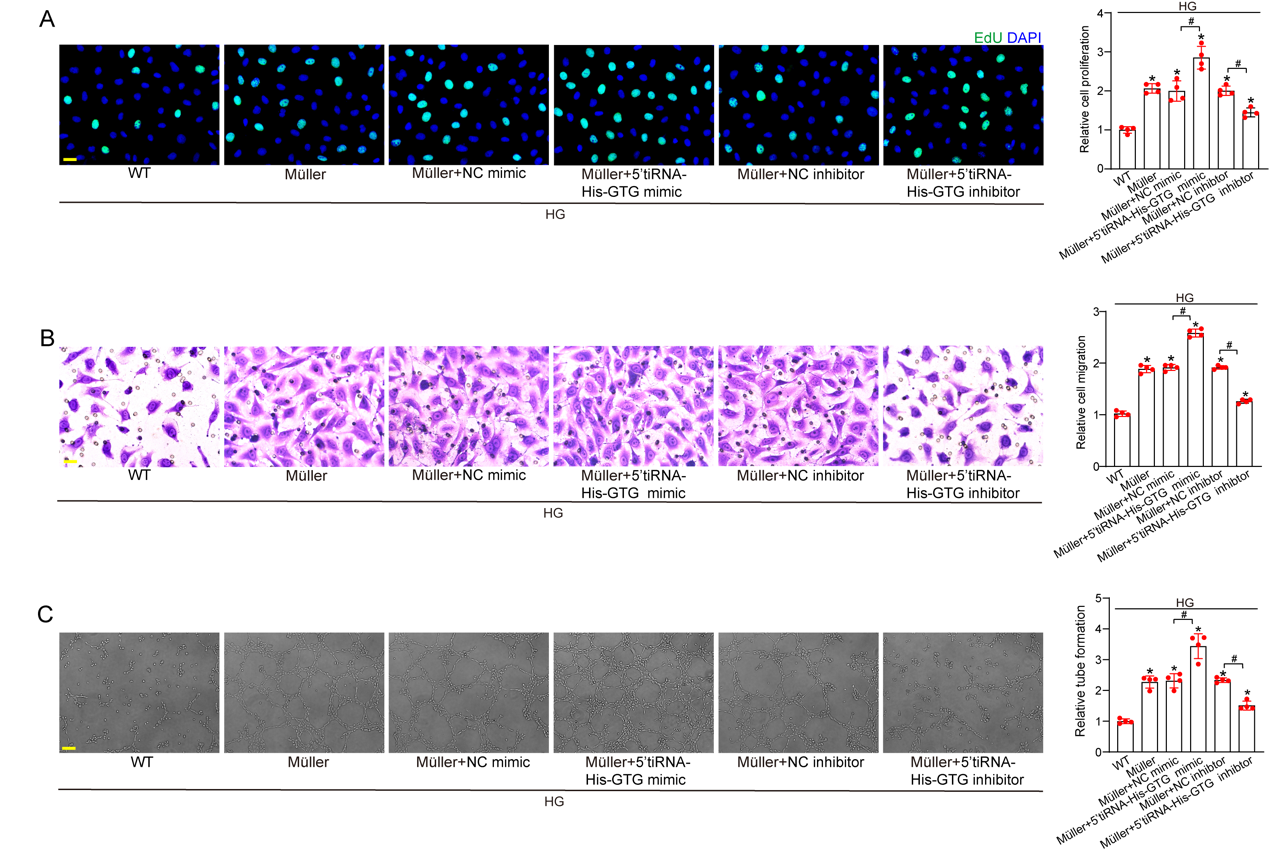


**Figure S2. The effect of 5’tiRNA-His-GTG on Müller cell-EC crosstalk *in vitro***

(A - C) HRMECs were co-cultured without or with Müller cells transfected with negative control (NC) mimic, 5’tiRNA-His-GTG mimic, NC inhibitor, 5’tiRNA-His-GTG inhibitor, or left untreated (WT), and then exposed to high glucose (30 mM, HG) for 48 h. Cell proliferation was examined by EdU staining after 48 h co-culture. EdU, green; DAPI, blue. Scale bar, 20 μm (A, n = 4). The migration ability of HRMECs was examined by transwell assays after 24 h co-culture. Scale bar, 20 μm (B, n = 4). The tube formation ability of HRMECs was observed at 14 h following cells seeding on the matrix. The average length of tube formation for each field was statistically analyzed. Scale bar, 100 μm (C, n = 4). **P* < 0.05 versus WT; ^#^*P* < 0.05 between the marked groups. The significant difference was evaluated by one-way ANOVA followed by post hoc Bonferroni test.


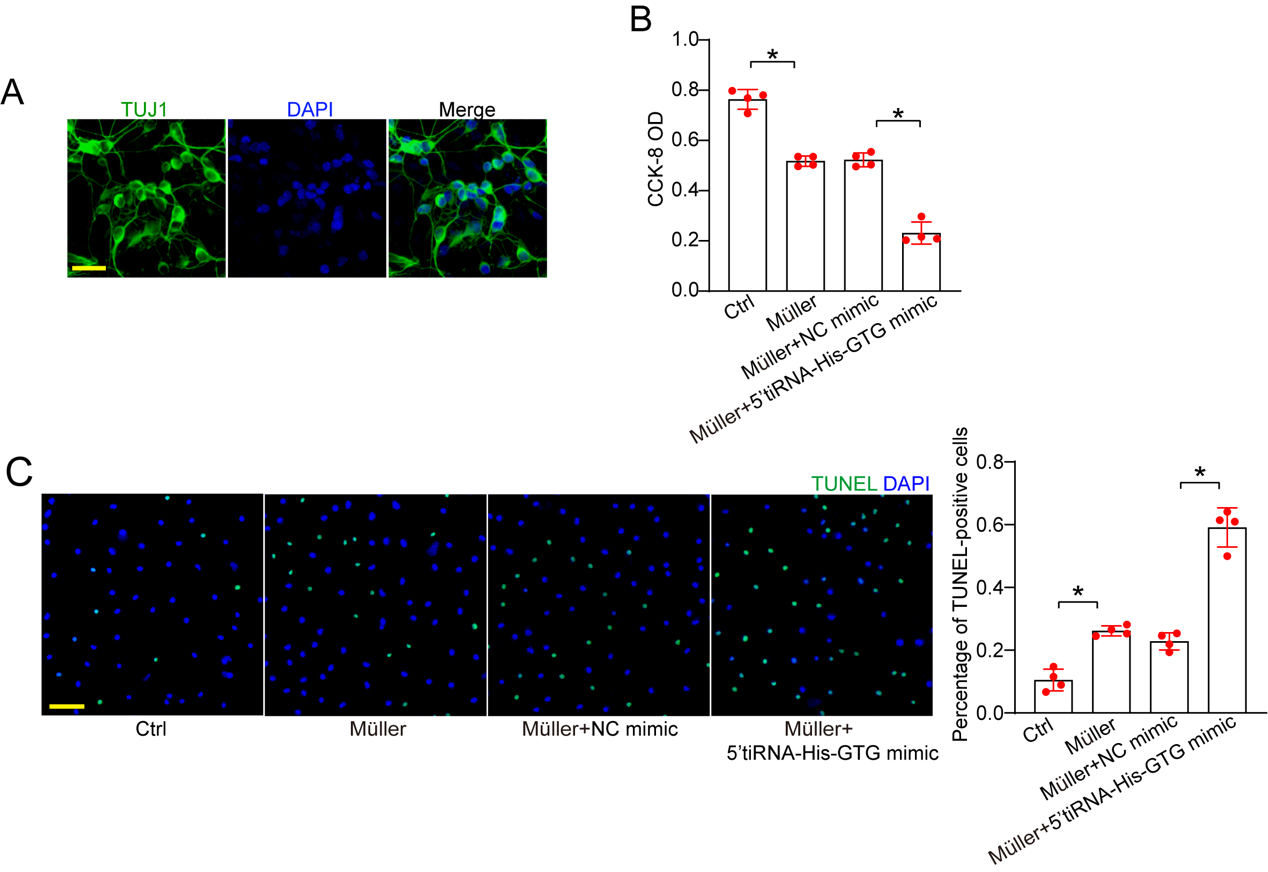


**Figure S3. The effect of 5’tiRNA-His-GTG on Müller cell-RGC crosstalk *in vitro***

(A) Primary RGCs were isolated from C57BL/6 mouse pups at postnatal day 0-3. Immunostaining of primary RGCs was conducted using TUJ1 antibody. TUJ1, green; DAPI, blue. Scale bar, 20 μm. (B and C) Primary RGCs were co-cultured without or with Müller cells after the transfection of negative control (NC) mimic, 5’tiRNA-His-GTG mimic, or left untreated (Ctrl), and then exposed to high glucose (30 mM, HG) for 24 h. The viability of primary RGCs was examined by CCK-8 assays (B, n = 4). Cell apoptosis was examined by TUNEL assays. TUNEL, green; DAPI, blue. Scale bar, 50 μm (C, n = 4). **P* < 0.05 between the marked groups. The significant difference was evaluated by one-way ANOVA followed by post hoc Bonferroni test.


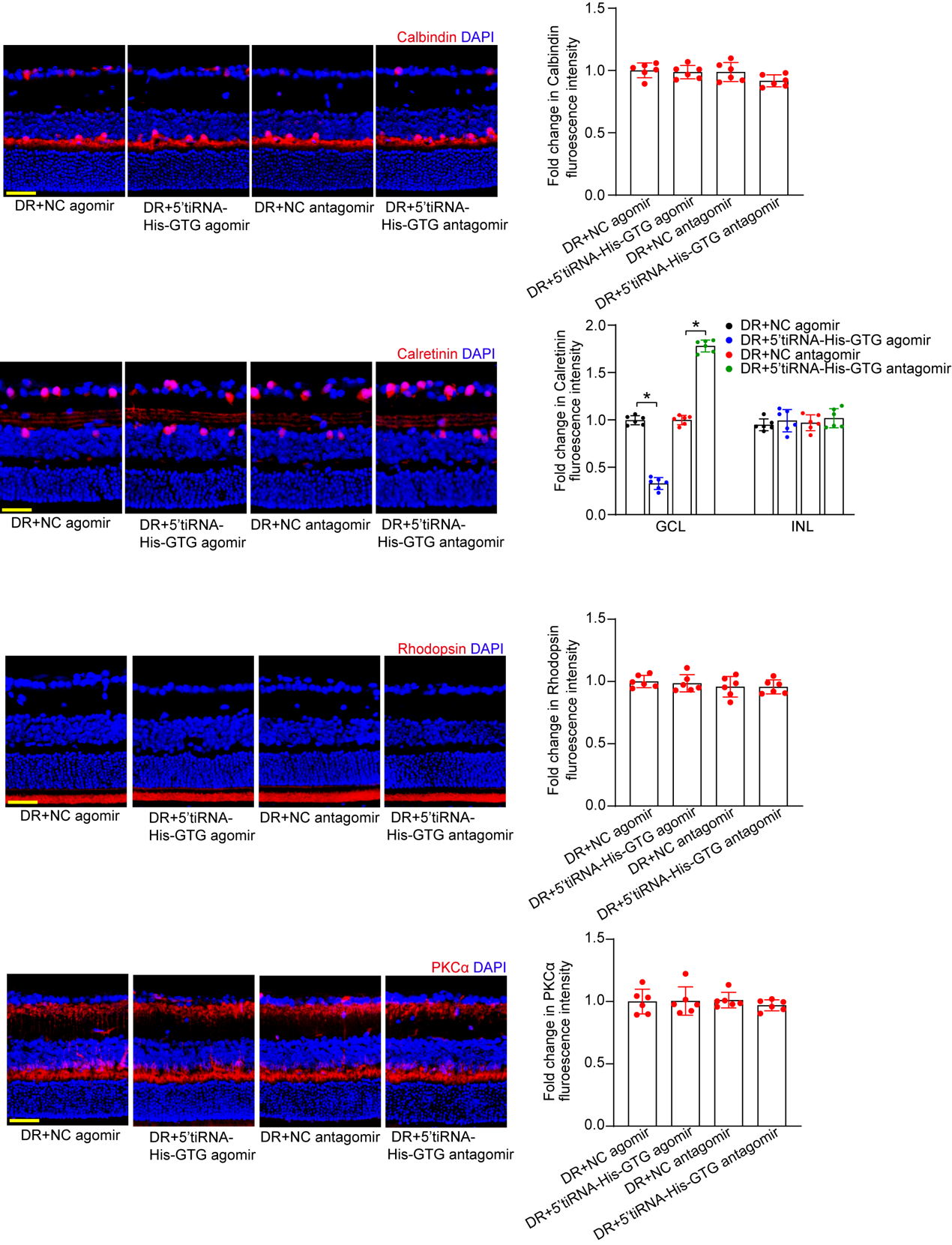


**Figure S4. 5’tiRNA-His-GTG has no effect on amacrine cells, horizontal cells, rod and cone photoreceptors, and bipolar cells in diabetic mice**

C57BL/6J mice received intravitreal injections of negative control (NC) agomir, 5’tiRNA-His-GTG agomir, NC antagomir, or 5’tiRNA-His-GTG antagomir following 4-month diabetes induction. Immunofluorescence stainings of calbindin, calretinin, rhodopsin, and PKCα were conducted to label amacrine cells, horizontal cells, rod and cone photoreceptors, and bipolar cells. The representative images and quantification results were shown (n = 6). Scale bar, 50 μm. **P* < 0.05 between the marked groups. The significant difference was evaluated by one-way ANOVA followed by post hoc Bonferroni test.

**Table S1. General physiological parameters of diabetic mice**

|  | Initial | | 2 months | | | 4 months | | |
| --- | --- | --- | --- | --- | --- | --- | --- | --- |
| Group | Body weight | Glucose | | Body weight | Glucose | | Body weight | Glucose |
|  | （g） | （mmol/L） | | （g） | （mmol/L） | | （g） | （mmol/L） |
| Diabetic+NC agomir | 24.95 ± 0.26 | 5.50 ± 0.17 | | 23.38 ± 0.29 | 24.25 ± 0.60* | | 23.65 ± 0.28 | 25.84 ± 0.61* |
| Diabetic+5’tiRNA-HIS-GTG agomir | 24.58 ± 0.26 | 5.42 ± 0.19 | | 23.34 ± 0.27 | 24.51 ± 0.54* | | 23.74 ± 0.37 | 26.36 ± 0.52* |
| Diabetic+NC antagomir | 25.21 ± 0.23 | 5.69 ± 0.19 | | 23.75 ± 0.30 | 25.77 ± 0.60* | | 23.44 ± 0.29 | 25.29 ± 0.46* |
| Diabetic+5’tiRNA-HIS-GTG antagomir | 24.42 ± 0.28 | 5.38 ± 0.17 | | 23.27 ± 0.31 | 25.03 ± 0.61* | | 23.27 ± 0.28 | 25.07 ± 0.59* |

All data were shown as mean ± SEM. The difference in physiological parameters between non-diabetic and diabetic mice was determined by repeated measures ANOVA at different time points. **P* < 0.05 compared with the corresponding initial group.

**Table S2. Baseline characteristics of the patients for fibrovascular membrane collection**

|  | Proliferative diabetic retinopathy  (n = 10) | Idiopathic epiretinal membranes  (n = 10) | *P* value |
| --- | --- | --- | --- |
| Age, y | 54.7 ± 2.2 | 55.1 ± 1.7 | 0.89 |
| Male, % | 50 | 50 | 1 |
| Total cholesterol, mmol/L | 4.83 ± 0.38 | 5.16 ± 0.59 | 0.64 |
| Creatinine, μmol/L | 84.50 ± 11.29 | 56.60 ± 4.13 | 0.03 |
| Triglyceride, mmol/L | 1.79 ± 0.35 | 1.11 ± 0.15 | 0.05 |
| HbAlc, % | 7.03 ± 0.38 | 5.76 ± 0.17 | 0.01 |

Data were expressed as mean ± SEM or percentage. *P* values were determined by Student’s *t* test or Fisher exact test.

| **Table S3. Clinical characteristics of patients for aqueous humor collection** | | | | | |
| --- | --- | --- | --- | --- | --- |
|  | DR(n=10) | Cataract(n=10) | | Trauma(n=10) | |
|  | number | number | *P* | number | *P* |
| Age, y | 53.1 ± 2.6 | 56.6 ± 2.8 | 1 | 48.1 ± 3.5 | 1 |
| Male, % | 60 | 50 | 0.653 | 70 | 0.639 |
| Total cholesterol, mmol/L | 4.28 ± 0.39 | 4.91 ± 0.37 | 1 | 4.22 ± 0.30 | 1 |
| Creatinine, μmol/L | 61.00 ± 6.06 | 60.10 ± 4.51 | 1 | 58.10 ± 2.14 | 0.998 |
| Triglyceride, mmol/L | 2.07 ± 0.35 | 1.56 ± 0.23 | 0.853 | 0.93 ± 0.17 | 0.013 |
| HbAlc, % | 8.14 ± 0.43 | 5.87 ± 0.17 | 0.002 | 6.28 ± 0.36 | 0.016 |
| Data were expressed as mean ± SEM or percentage. *P* value was determined by one-way ANOVA test or Fisher exact test, and calculated by comparing with DR group. | | | | | |

| **Table S4. RNA oligonucleotide sequences** | |
| --- | --- |
| Name | Sequence (5'-3') |
| 5'tiRNA-His-GTG mimic | GCCGUGAUCGUAUAGUGGUUAGUACUCUGCGUUGU |
| 5'tiRNA-His-GTG inhibitor | ACAACGCAGAGUACUAACCACUAUACGAUCACGGC |
| NC mimic | UUGUACUACACAAAAGUACUG |
| NC inhibitor | CAGUACUUUUGUGUAGUACAA |
| 5'tiRNA-His-GTG agomir | GCCGUGAUCGUAUAGUGGUUAGUACUCUGCGUUGU |
| 5'tiRNA-His-GTG antagomir | ACAACGCAGAGUACUAACCACUAUACGAUCACGGC |
| NC agomir | UUGUACUACACAAAAGUACUG |
| NC antagomir | CAGUACUUUUGUGUAGUACAAA |

| **Table S5. Primer sequences for qPCR assays** | | | | |
| --- | --- | --- | --- | --- |
| Primer sequence | | | | |
| 5'tiRNA-His-GTG | | Forward |  | 5’-ATCGCCGTGATCGTATAGTGG-3’ |
|  |  | Reverse |  | 5’-CTTCCGATCTACAACGCAGAGTAC-3’ |
| U6 | | Forward |  | 5'-GCTTCGGCAGCACATATACTAAAAT-3' |
|  |  | Reverse |  | 5'-CGCTTCACGAATTTGCGTGTCAT-3' |
| ANG | | Forward |  | 5’-AGAAGCGGGTGAGAAACAAAAC-3’ |
|  |  | Reverse |  | 5'-AGTGCTGGGTCAGGAAGTGTG-3' |
| β-actin | | Forward |  | 5’-CACCATTGGCAATGAGCGGTTC-3’ |
|  |  | Reverse |  | 5’-AGGTCTTTGCGGATGTCCACGT-3’ |

**Supplemental materials and methods**

**Cell isolation and culture**

Human retinal Müller cells were sampled from two patients undergoing traumatic enucleation without any known history of ocular diseases. Primary mouse Müller cells were obtained from the postnatal day 11-12 mice. The retinas were removed from the posterior eyeballs and placed in DMEM medium on ice. The dissected retinas were minced with a micro-scissor. Retinal tissues were dissociated using the Worthington-Papain Dissociation System and filtered through a 30-μm nylon strainer. After 7 to 12-day culture, retinal cells were treated with 0.05% Trypsin-EDTA for 1 min at 37°C to isolate the adherent microglia. The purity was identified by indirect fluorescent immunolabelling with glial acidic fibrillary protein (GFAP) and glutamine syntheses (GS). Primary Müller cells were cultured in the medium containing DMEM, 1% GlutaMax, 1% penicillin/streptomycin, and 10% FBS. Cells between passages 2 and 5 were used for subsequent experiments.

The two-step immunostaining protocol was followed to undertake the procedures for isolating and culturing mouse primary RGCs. In brief, retinas were obtained from C57BL/6J mouse pups on postnatal day 1-3 and then dissociated in 15 U/ml papain and 70 U/ml collagenase in PBS solution for 15 min. After that, retinal cell suspensions were treated with flasks that had been coated with anti-macrophage antiserum antibody (1:100; AIA31240; Accurate Chemical & Scientific Corporation) to eliminate adhering macrophages and microglial cells. To isolate and purify mouse primary RGCs, non-adherent cells were first treated with Thy1.2 monoclonal antibody (MCA02R; Bio-Rad). After dissociation with 0.025% trypsin (Sigma, T9201, USA), primary RGCs were seeded at a density of 1.8 × 10^5^ cells per well in a 24-well plate that had been precoated with 50 mg/mL poly-L-lysine-coated flasks. The purity of RGCs was determined by staining with TUJ1 antibody (ab18207, Abcam, USA). Primary RGCs were cultured in serum-free Neurobasal-A media (Gibco) supplemented with 1% B27 (Gibco), 1% glutamine (Invitrogen), 5 mM of D-glucose, and 50 U/mL of penicillin-streptomycin.

Human retinal microvascular endothelial cells (HRMECs) and retinal pericytes were obtained from Cell Systems. ARPE-19 cells were purchased from Amercian Type Culture Collection (ATCC). HRMECs were cultured in endothelial growth medium (EGM2-MV) supplemented with 5% FBS. Pericytes were cultured in DMEM medium containing 10% FBS. ARPE-19 cells were maintained in DMEM-F12 medium with 10% FBS. All cells were cultured in a humidified incubator containing 5% CO_2_ incubator at 37 °C.

**Cell counting Kit-8 (CCK-8) assay**

CCK-8 assay (Beyotime, C0038) was conducted to assess cell viability as per the manufacturer’s instructions. After the required treatment, cells were planted at a density of 2 × 10^4^ per well in 96-well plates. Each well was loaded with 10% CCK-8 reagent and incubated at 37°C in the dark. The optical density was measured at the wavelength of 450 nm using a microplate reader.

**TdT-mediated dUTP nick-end labeling (TUNEL) assay**

TUNEL assays were performed according to the manufacturer’s protocol (Roche, 12156792910). Briefly, cells were planted into the 24-well cell-culture plates. Following high glucose treatment, the TUNEL *In Situ* Cell Death Detection Kit was used to assess cell apoptosis. TUNEL (5 μM) was used to stain cells and DAPI was used to stain cell nuclei. To determine TUNEL ratio (% vs. DAPI), TUNEL-positive cells from at least five different random fields were counted for each treatment using an Olympus IX-73 fluorescence microscopy.

**Tube formation assay**

HRMECs (2 × 10^5^/well) were plated out onto 24-well plates that had previously been coated with basement membrane matrix (Coring, 356234) and incubated in the indicated conditioned medium. HRMECs were maintained in an incubator that was humidified and supplied with 5% CO_2_. The Olympus IX-73 microscope was used to observe and acquire the images of tubular structures. The average total length of tube formation for each field was statistically analyzed with Angiogenesis Analyzer for Image J software.

**Transwell assay**

Transwell assays were performed using a Transwell insert (Millipore, PTEP24H48) with an 8-μm pore size. HRMECs（1×10^5^ cells/well）were added to the upper chamber filled with 100 μl of serum-free culture media. The lower chamber's base was filled with 600 μl DMEM with 10% FBS. After incubation, transwell chambers were preserved with 20% methanol for 15 min before being stained with 0.1% crystal violet at room temperature for 20 min. After that, cells were carefully removed off the upper side of the membranes with a cotton swab. A light microscope was used to randomly count the cells that moved to the chambers' bottom. Relative migration values were calculated as the number of cells that had migrated across a transwell plate compared to the control group.

**Rhodamine 123 staining**

Rhodamine 123 staining was used to detect mitochondrial membrane potential. Rhodamine 123 is a cationic fluorescent indicator that selectively accumulates within mitochondria in a membrane potential-dependent way. Following transfection and high glucose treatment, cells were harvested with Rhodamine 123 (10 μM, Solarbio, R8030) for 15 min at 37°C in the dark and washed three times by PBS. The decrease in green rhodamine 123 fluorescence indicates the dispersion of ΔΨm. Images were captured using an Olympus IX-73 microscope and measured using Image J software.

**Calcein-AM and propidium iodide (PI) double staining**

PI/Calcein-AM double staining kit was used to detect cell apoptosis. Calcein-AM is permeable to cell membrane and stains living cells, whereas PI is impermeable to the cell membrane and stains dead cells. Briefly, the cells were harvested and stained with 10 μM of Calcein-AM (AAT Bioquest, 22002) and PI (Biofroxx, 1246MG100) solution for 20 min at 37 °C in the dark. The images were acquired by Olympus IX-73 microscope. Live cells were viewed using an excitation filter of 490 nm, while dead cells were observed with an excitation filter of 545 nm. The number of stained cells in at least five randomly selected fields was determined.

**RNA extraction and quantitative reverse transcription-PCR (qRT-PCR)**

TRIzol reagent (Life Technologies, 15596026) was used to isolate total RNAs from cells, retinas, and clinical samples. The quality and purity of total RNAs were detected by spectrophotometry. A Cytoplasmic & Nuclear RNA Purification Kit (Norgen Biotek, NGB-21000) was used to separate RNAs from the nucleus and cytoplasm. The rtStar^TM^ First-strand cDNA Synthesis kit (Arraystar, AS-FS-003) and SuperScript IV First-Strand Synthesis System (Thermo Fisher Scientific, 18091050) were used to synthesize cDNAs from 5'tiRNA-His-GTG and other genes. qRT-PCRs were conducted using SYBR Green Master Mix (Thermo Fisher Scientific, 100029284). The PikoReal Real-Time PCR System (Thermo Scientific) was used to conduct qRT-PCRs. The expression levels of 5'tiRNA-His-GTG were normalized relative to U6, and the relative expression levels of other mRNA were normalized relative to β-action.

Western blot

Treated Müller cells and retina tissues were lysed using RIPA buffer containing Complete™ protease inhibitor cocktail (Roche, 04693132001). After centrifugation at 12, 000 *g* for 20 min at 4 ℃, the supernatants were kept for protein measurement. QuantiPro™ BCA Assay Kit (Thermo, 23227) was used to determine protein concentration. All protein samples were run on SDS-PAGE gels and transferred onto

the polyvinylidene fluoride (PVDF) membranes (Millipore, ISEQ00010). After blocking with 5% bovine serum albumin (BSA) for 1 h, the membranes were incubated with the primary antibody overnight at 4°C. β-actin was detected as the internal control. Then, the membranes were incubated with the horseradish peroxidase (HRP)-conjugated secondary antibody (Beyotime) for 2 h at room temperature. The bands were detected with the enhanced chemiluminescence system (Amersham Biosciences). The intensities of protein bands were scanned and quantified using the Quantity One software (Bio-Rad, VA, USA).

Intravitreal injection

The mice were anesthetized with an intraperitoneal injection of the mixture of ketamine (80 mg/kg) and xylazine (10 mg/kg). The following reagents were administered by intravitreal injection: NC agomir, 5'tiRNA-His-GTG agomir, NC antagomir, or 5'tiRNA-His-GTG antagomir. 1% tropicamide was applied topically to dilate the pupils of the mice. To avoid injuries to ocular tissues, the injections were performed twice a month using a 33-gauge needle Hamilton micro syringe, which is injected at a 45° injection angle through the sclera into vitreous body. Ofloxacin ointment (Santen, Osaka, Japan) was used for preventing bacterial infection.

Immunohistochemistry

Eyecups were cryoprotected in 30% sucrose overnight at 4°C and then they were embedded in OCT compound. Embedded eyecups were sectioned by cryostat at 10 μm thickness and thaw-mounted onto poly-L-lysine coated slides. Retinal sections were blocked and permeabilized in 1% BSA and 0.5% Triton X-100 for 1 h at 37°C. Then, the sections were incubated with primary antibodies at 4°C overnight: GFAP (1:200, Abcam, ab68428), Vimentin (1:300, Abcam, ab8978), TUJ1 (1:200, Abcam, ab18207), NeuN (1:300, Abcam, ab177487), Calbindin (1:200, Santa Cruz Biotechnology, sc-365360), Calretinin (1:500, Santa Cruz Biotechnology, sc-365956), Rhodopsin (1:400, Abcam, ab5417) and PKCα (1:400, Abcam, ab32376). Retinal sections were washed three times in PBS and then incubated in Alexa-Fluor-488 and Alexa-Fluor-594 (both Invitrogen) conjugated secondary antibodies for 2 h at room temperature. Images of immunohistochemical staining were examined by an Olympus IX-73 microscopy and analyzed by Image J.

Whole-mounted retinal immunofluorescence

The neural retina survival rate was determined by immunostaining the whole-mounted retinas with TUJ1 antibody (an RGC marker). The eyes were incubated in 4% PFA for 30 min at room temperature. The retinas were separated from the sclera and flattened with four incisions as a petal shape on a glass slide. After being blocked and permeabilized overnight at 4 °C in a buffer containing 0.2% Triton-X-100, the whole retinas were incubated for 1 h with 5% BSA dissolved in PBS. Then, the retinas were incubated with anti-TUJ1(1:400, Abcam, ab18207) overnight at 4 °C, followed by 2 h at room temperature with secondary antibodies. After retinas were mounted in an anti-fade solution, the Images were taken using an Olympus IX-73 fluorescence microscope. Image J was used to count TUJ1^+^ cells, and the number of surviving RGCs was compared with the control group to determine RGC survival.
